# PyCogent: a toolkit for making sense from sequence

**DOI:** 10.1186/gb-2007-8-8-r171

**Published:** 2007-08-21

**Authors:** Rob Knight, Peter Maxwell, Amanda Birmingham, Jason Carnes, J Gregory Caporaso, Brett C Easton, Michael Eaton, Micah Hamady, Helen Lindsay, Zongzhi Liu, Catherine Lozupone, Daniel McDonald, Michael Robeson, Raymond Sammut, Sandra Smit, Matthew J Wakefield, Jeremy Widmann, Shandy Wikman, Stephanie Wilson, Hua Ying, Gavin A Huttley

**Affiliations:** 1 Department of Chemistry and Biochemistry, University of Colorado, Boulder, Colorado, USA; 2 Computational Genomics Laboratory, John Curtin School of Medical Research, The Australian National University, Canberra, Australian Capital Territory, Australia; 3 Thermo Fisher Scientific, Lafayette, Colorado, USA; 4 Seattle Biomedical Research Institute, Seattle, Washington, USA; 5 Department of Biochemistry and Molecular Genetics, University of Colorado Health Sciences Center, Aurora, Colorado, USA; 6 Science Applications International Corporation, Englewood, Colorado, USA; 7 Department of Computer Science, University of Colorado, Boulder, Colorado, USA; 8 Department of Ecology and Evolutionary Biology, University of Colorado, Boulder, Colorado, USA; 9 Walter and Eliza Hall Institute, Melbourne, Victoria, Australia

## Abstract

We have implemented in Python the COmparative GENomic Toolkit, a fully integrated and thoroughly tested framework for novel probabilistic analyses of biological sequences, devising workflows, and generating publication quality graphics. PyCogent includes connectors to remote databases, built-in generalized probabilistic techniques for working with biological sequences, and controllers for third-party applications. The toolkit takes advantage of parallel architectures and runs on a range of hardware and operating systems, and is available under the general public license from .

## Abstract 2

The COmparative GENomic Toolkit, a framework for probabilistic analyses of biological sequences, devising workflows and generating publication quality graphics, has been implemented in Python.

## Rationale

The genetic divergence of species is affected by both DNA metabolic processes and natural selection. Processes contributing to genetic variation that are undetectable with intra-specific data may be detectable by inter-specific analyses because of the accumulation of signal over evolutionary time scales. As a consequence of the greater statistical power, there is interest in applying comparative analyses to address an increasing number and diversity of problems, in particular analyses that integrate sequence and phenotype. Significant barriers that hinder the extension of comparative analyses to exploit genome indexed phenotypic data include the narrow focus of most analytical tools, and the diverse array of data sources, formats, and tools available.

Theoretically coherent integrative analyses can be conducted by combining probabilistic models of different aspects of genotype. Probabilistic models of sequence change underlie many core bioinformatics tasks, including similarity search, sequence alignment, phylogenetic inference, and ancestral state reconstruction. Probabilistic models allow usage of likelihood inference, a powerful approach from statistics, to establish the significance of differences in support of competing hypotheses. Linking different analyses through a shared and explicit probabilistic model of sequence change is thus extremely valuable, and provides a basis for generalizing analyses to more complex models of evolution (for example, to incorporate dependence between sites). Numerous studies have established how biological factors representing metabolic or selective influences can be represented in substitution models as specific parameters that affect rates of interchange between sequence motifs or the spatial occurrence of such rates [1-4]. Given this solid grounding, it is desirable to have a toolkit that allows flexible parameterization of probabilistic models and interchange of appropriate modules.

There are many existing software packages that can manipulate biological sequences and structures, but few allow specification of both truly novel statistical models and detailed workflow control for genome scale datasets. Traditional phylogenetic analysis applications [5,6] typically provide a number of explicitly defined statistical models that are difficult to modify. One exception in which the parameterization of entirely novel substitution models was possible is PyEvolve [1], which has been incorporated as part of PyCogent and significantly extended. However, building a phylogenetic tree is only one step in most comparative genomics workflows. Specifying a workflow requires connecting to raw data sources, conducting or controlling specific analytical processes, and converting data between different components. Such tasks are achievable to differing degrees using the Bio-language (for example, BioPerl [7] and BioPython [8]) libraries, although these projects are limited in their built-in evolutionary modeling capabilities. Finally, support by either analysis or workflow applications for visualization, which is a critical aspect of data analysis, is often limited. Workflow tools such as CIPRES/Kepler [9] allow flexible connection of pre-coded components within the context of a graphical user interface, but they have (at least at the time of writing) a relatively small range of built-in analyses. We list a summary of features from several comparative genomics tools in Table 1.

**Table 1:** Summary of features of selected comparative genomics tools

Feature	PyCogent	HyPHY	P4	BioPython	Mesquite	ARB	CIPRES^a^
Query remote database	Yes	No	No	Yes	Yes	Yes	No
Control external	Yes	No	No	Yes	Yes	Yes	Yes
Create novel substitution	Yes	Yes	Yes	No	Yes	No	No
Novel sequence alignment	Yes	No	No	Yes	No	No	No
Partition models	Yes	Yes	No	No	No	No	No
Slice sequences	Yes	No	No	Yes	No	No	No
Draw alignments	Yes	No	No	No	No	No	No
Build phylogenetic trees	Yes	Yes	Yes	No	Yes	Yes	Yes
Draw phylogenetic trees	Yes	Yes	Yes	No	Yes	Yes	Yes
Visualization of model	Yes	Yes	No	No	Yes	No	No
Parallel computation	Yes	Yes	No	Yes	No	No	Yes
Customize parallelization	Yes	Yes	No	Yes	No	No	Yes
Reconstruct ancestral	Yes	Yes	No	No	Yes	Yes	Yes
Simulate sequences	Yes	Yes	Yes	No	Yes	No	Yes
Graphical user interface	No	Yes	No	No	Yes	Yes	Yes
Script based control	Yes	Yes	Yes	Yes	Yes	No	Yes
Handle 3D structures	Yes	No	No	Yes	Yes	Yes	No
Handling RNA secondary	Yes	No	No	No	No	Yes	No

PyCogent provides a unique combination of evolutionary modeling capabilities, visualization, and workflow control. Although many packages, including but not limited to those shown, provide some overlap in capabilities, PyCogent provides a combination of features that is uniquely suited to genome-scale analyses. ^a^CIPRES requires purchase of commercially licensed software for core functionality. 3D, three-dimensional.

Here, we describe PyCogent, a toolkit designed for connecting to databases, implementing novel analyses, controlling workflows, and visualization. Below, we outline some of the toolkit's capabilities and demonstrate them through three specific comparative genomics case studies. We take the opportunity to emphasize here that PyCogent has capabilities that are of value in other biological fields. The toolkit includes many functions of generic utility, such as simplified reading and manipulation of tabular data, and common statistical functions. Also included are facilities to expand the capabilities of PyCogent beyond the provided selections of data format parsers, database connectors, and third-party application controllers. We have applied several of these capabilities in population genomics studies, but their potential utility spans a wide range of bioinformatics and genomics tasks. The software is available online [10].

In the remainder of this report we use monospace type to distinguish literal code and arguments.

## Features and capabilities

### Design

The design of PyCogent was motivated by specific criteria arising from the analysis of other packages that were available at the time when development began, but that lacked the advanced modeling and visualization capabilities we needed. The main goal was to take a page from Perl's book, following Larry Wall's often quoted dictum: 'easy things should be easy, and hard things should be possible'. Easy things that should be easy include reading standard formats from a file, running simple phylogenetic analyses and alignments from the toolkit's repertoire or using standard third-party applications such as ClustalW [11] or basic local alignment search tool (BLAST) [12], retrieving sequences by ID from different databases, coloring important features on trees, alignments and crystal structures, and calculating statistics on sequences and alignments. Hard things that should be possible include parallel phylogenetic analyses of large sequences or many sequences, exotic substitution models for phylogenetic analyses or alignments, and the coordination of complex bioinformatics pipelines distributed across a network.

Our design criteria thus included the following general features (Additional data file 1 provides more detailed descriptions of functionality):

1. maintainable, reliable, and as platform-independent as possible;

2. self-contained, with relatively few, and optional, external dependencies;

3. clean, consistent application programming interface (API), allowing operations to be performed in few steps with limited syntax, ideally in an interactive shell;

4. flexible framework for adding new functionality, such as evolutionary models, parsers, and so on;

5. seamless integration with a large number of widely used existing tools, for example command-line applications such as BLAST, external databases, and external high-performance code such as tuned BLAS (basic linear algebra subprograms) libraries [13];

6. high-performance code base (genomics datasets tend to be large, and so easy implementation of parallel computation is important);

7. comprehensive and up-to-date user documentation;

8. combined code and documentation files to make it clear how to modify canned analyses (executable documentation stays up-to-date because tests fail if changes break the documentation); and

9. advanced visualization capabilities, including graphical reports for common annotation, alignment, phylogeny, and structure tasks.

### Implementation

We chose to implement PyCogent in the Python programming language for four main reasons. First, Python is an object-oriented programming language that allows a mixed model of procedural, object-oriented, and functional programming, and thus it provides a choice of styles to suit specific bioinformatics tasks. For example, functional programming techniques such as the map() and reduce() built-in functions, and the ability to create functions on the fly and use them as first-class objects, are extremely useful. The Alignment object provides a good example of the utility of these techniques. It is often useful to mark or eliminate sequences that meet specific user-defined criteria, such as the presence or absence of specific motifs. This can be accomplished by creating a function that tests a single sequence for the presence of the motif, mapping the rows of the alignment to boolean values based on the result of this function, and then filtering the alignment based on the value of the test for each row.

Second, Python is increasingly gaining currency in the scientific programming and high-performance computing communities, with mature third-party libraries such as Numpy [14], ReportLab [15], and Matplotlib [16] providing important numerical and reporting capabilities. This popularity in part reflects the relative ease of teaching scientists who are casual programmers the lightweight syntax needed to get started with Python.

Third, Python's introspection capabilities, and advanced interactive shells such as ipython [17], make the language well suited to analysis of exploratory data, especially because the capabilities of objects can be interrogated on the fly with built-in functions such as dir().

Fourth, Python is easily extensible with tools such as Pyrex [18], which allows performance critical tasks to be coded in a Python-like language that compiles with C extensions. This extensibility, along with Python's comprehensive built-in profiling and unit testing tools, allows attention to be focused first on the correctness of the code and second to identify and correct bottlenecks, in line with Hoare's dictum, 'Premature optimization is the root of all evil'.

Finally, Python's built-in doctest module allows testing of Python code embedded in standard text documents, delimited solely by the Python interactive interpreter's command input ('>>>') and continuation ('...') symbols, to be executed and tested. This doctest module allows a user to produce computable documents, such that the accuracy of the documentation can be assessed; it also provides a means for using Python to achieve the goals of reproducible computational research [19,20]. If the code that performs the analysis and the associated data files are distributed as part of an executable publication, then other researchers can run the code and verify that the same (or, for stochastic analyses, qualitatively similar) results are obtained. PyCogent supplements its formal unit tests for each module with standalone doctest documents that combine the explanation of specific analyses with computable code to run those analyses.

It has been observed that the most successful open source projects, such as the Linux kernel and the Apache web server, rely on tight control of contributions by a small development team. This tiered model of development allows many contributions and bug fixes to be considered, but restricted access for committing changes allows the project to keep a coherent style and API. Accordingly, with PyCogent we have opted for an open source license (the general public license), but contributions are peer reviewed and edited before being accepted. New features or significant modifications are always accompanied by both unit tests and integration tests that are automatically triggered by commits to the repository; if tests fail, then the contribution is rejected. This restriction ensures that the core PyCogent library is always in a working state. Strict naming guidelines for classes, functions, methods, abbreviations, and so on, described in the PyCogent documentation, are enforced to facilitate discovery of the API's features. This API consistency eases interactive analyses considerably, because the abbreviation and capitalization of names can usually be guessed rather than remembered, reducing the cognitive burden on the user. The majority of documentation for PyCogent is written as standalone doctests, and the validity of the documentation is checked before releases. Features and APIs that are deprecated must first pass through a warning stage before they may be removed from the toolkit, allowing sufficient time for modification of legacy code.

### Key features of PyCogent

PyCogent consists of 17 top-level packages (Additional data file 1), which provide core objects for representing and manipulating biological data, mathematical and statistical functionality, parsers for a wide range of bioinformatics file formats, and code for manipulating external databases and applications. An overview of each package for developers can be found in Additional data file 1, and the toolkit is distributed with extensive examples that illustrate usage. Here, we discuss some of the design considerations and features that support common bioinformatics tasks.

#### Querying databases

Most bioinformatics analyses begin by obtaining information from public databases. Our goal for database access was to minimize the overhead involved in querying or in obtaining records by accession. In general, our database accessors behave either like dictionaries keyed by the accession number of each sequence or like containers that return query results.

For example, to retrieve a sequence from GenBank, the following code suffices:

>>> from cogent.db.ncbi import EUtils

>>> e = EUtils()                     

>>> result = e['AF0001']             

By default, the result of the EUtils (Entrez Utilities) call will be an open FASTA format file, and the nucleotide database will be searched. Arbitrary queries can also be handled in the same interface. For example, we can retrieve the first 100 bacterial lysyl-tRNA synthetase protein sequences in GenBank format as follows:

>>> e = EUtils(numseqs=100, db='protein', rettype='genpept')    

>>> result = e['"lysyl tRNA-synthetase"[ti] AND bacteria[orgn]']

The goal of the database adaptors is to make simple queries as efficient as possible in terms of both usability and computational performance, and to allow re-use of the syntax familiar from the web query interface of each database. Fine-grained control over parameters such as the number of records returned in each set and the wait time between queries is also available.

Adaptors for querying National Center for Biotechnology Information (NCBI; including PubMed), Protein Data Bank (PDB), and RNA families database (Rfam) are currently available, along with a framework that facilitates rapid development of additional controllers.

#### Handling biological data

The core objects in PyCogent include facilities for handling sequences, alignments, trees, compositions (for example, codon usage or amino acid usage), and annotations. Again, the primary goal has been to simplify file input/output as much as possible while allowing fine-grained control over the details for expert users. For example, parsing a GenBank file 'sequences.gbk' on disk can be as simple as follows:

>>> from cogent import LoadSeqs                   

>>> seqs = LoadSeqs('sequences.gbk', aligned=False)

In this case, the file type is automatically detected from the file extension and dispatched to the appropriate parser. The file format may also be specified using a format argument to the LoadSeqs function. Similarly, loading an alignment from a file (for example, in multi-FASTA format) is simply

>>> seqs = LoadSeqs('sequences.fasta')

Loading a tree from a file is equally straightforward, for example

>>> tree = LoadTree('mytree.tree')

If more control is required, then the individual parsers can be imported from their respective modules. Rather than using regular expressions to recognize and parse each record, which can be a fragile approach, we instead use a nested hierarchy of parsers. The parser at each level recognizes pieces of a record, and dispatches each piece to the appropriate lower level parser. The advantages of this approach are that unrecognized fields, such as those newly introduced into a database format, can be ignored rather than crashing the parser, and lightweight parsers that ignore all but a few selected pieces of the record can readily be generated. The hierarchical approach also ensures that only the lines of the file pertaining to the current record are kept in memory, so that very large files can be processed one record at a time. This incremental parsing can lead to large performance increases. Technically, the individual parsers are all generators that yield each record as it is read, although a list containing all records can easily be produced. A major advantage of the generator approach is that filtering can be applied on a per-record basis as the records are read, potentially providing large memory savings. For example, sequences could be filtered by length, by number of degenerate nucleotides, or by annotation.

The SequenceCollection and Alignment objects have several useful methods for filtering sequences by quality, converting between sequence types and alphabets, and so forth. For example, the Alignment can delete sequences that introduce gaps in other sequences, can eliminate columns of gaps or degenerate bases, or can delete sequences that are more than a specified distance from a template sequence. It can also convert between dense alignments, which store the alignment as an array of characters, and sparse alignments, which store the original sequences and a mapping between sequence and alignment coordinates. Dense alignments are useful for analyzing large numbers of sequences that contain few gaps (for instance, for individual protein families) and sparse alignments are useful for analyzing small numbers of sequences with many gaps (for instance, whole genome alignments).

Annotations are preserved in the alignment, allowing features on different sequences to be related to one another. Two types of annotations are handled: meta-data such as species, source, and citations, which are stored in an Info object that can be shared between different forms of a sequence (for example, the DNA, RNA, and corresponding protein); and annotation tracks, in which biological attributes that have sequence locations are explicitly represented as objects that can be applied to both sequences and alignments. It is frequently the case that for an analysis the user wishes to stratify by meta-data, such as genic, intergenic, or coding sequence (CDS). PyCogent's annotation tracks allow such slicing as illustrated in the case studies below.

The Tree object is implemented recursively, so that any fragment of a tree can be subjected to any analysis that works on the whole tree. Additionally, the tree objects contain code for deleting nodes by annotation (including pruning branches with single children), calculating distances between taxa, collapsing poorly resolved nodes, and evolving sequences according to a specified substitution model.

#### Built-in analyses

PyCogent supports many key evolutionary analyses directly, including sequence alignment, phylogenetic reconstruction, and model inference. These analyses are intended to be as flexible as possible, and to allow users to implement easily new capabilities that were not originally intended by the authors. This flexibility addresses a problem with many existing packages, which often provide a range of model parameterizations that cannot be extended. The core analyses are alignment (either using traditional approaches such as Smith-Waterman or using pair-hidden Markov models [HMMs]), flexible evolutionary modeling (see the first case study, below), ancestral sequence reconstruction, tree reconstruction, and sequence simulation. These analyses can be performed using arbitrarily complex evolutionary models. For example, nucleotide, codon, or amino acid models can be used for any of these tasks, and the parameterization of those models can be customized. One key advantage of this approach is that there is complete orthogonality between the models and the analyses, so that the effects of varying the model and varying the analysis method can be assessed independently. This independence is especially important when estimating the effects of violating the constraints of a given (unknown) model on the accuracy of an analysis. Additionally, models that allow dependence between adjacent sites, such as accounting for deamination in CpG dinucleotides, can be implemented and used in any of these analyses [1,2]. When these models are nested, likelihood ratio tests can easily be applied using supporting PyCogent infrastructure, as illustrated in the first case study (below).

#### Controlling third-party applications

One critical component of a bioinformatics toolkit is the ability to control third-party applications. This capability is essential for testing new methods against the existing state of the art, for coordinating large numbers of analyses on a parallel cluster, for performing sensitivity analysis, and for incorporating third-party applications into workflows. PyCogent has a generic application controller framework that encapsulates the code necessary for passing command-line parameters and reading multiple output files and streams. A phylogeny package such as RAxML [21], for instance, produces several different output files, and also prints information to the standard output and error streams. Invoking an application such as ClustalW [11] is straightforward:

>>> from cogent.app.clustalw import Clustalw

>>> app = Clustalw()                        

>>> result = app('sequences.fasta')['Align']

In addition to the alignment and guide tree, the exit status and error streams are also captured. Note that despite the simplicity of this example, the full set of parameters in the application is exposed in the interface, allowing fine-grained control of the analysis. Controllers are currently implemented for several other applications, including the following: the homology search packages BLAST [12], position-specific iterative BLAST [22], and BLAST-like alignment tool [23]; the alignment packages ClustalW [11], Muscle [24], and Dialign [25]; the motif finder MEME [26]; the phylogeny package RAxML [21]; and the structure analysis packages RNAView [27] and Vienna RNA [28].

#### Parallel computation support

PyCogent implements novel algorithms for parallelization of computational tasks at either the data or algorithmic levels. The parallel module convenience function localShareOf provides a mechanism for data based sharing of tasks across CPUs, while the output_cpu attribute simplifies the output of data by only one CPU. Algorithmic level parallelization is built into certain PyCogent components, primarily in the form of distributing across multiple CPUs, alignment positions for the likelihood calculations, or alternate topologies for the advanced stepwise addition tree topology search algorithm. Additional complexity in parallel computation is supported by the parallel module's implementation of a virtual CPU stack. Under this design, for instance, one could simultaneously employ both a data and algorithm level parallel strategy. For example, at the top virtual level two long alignments could assigned to separate dual core CPUs, whereas at the lower level their likelihood calculations may be split by position across the two cores. PyCogent also supports task farming of third-party applications, including activation of parallelism already used by those programs (such as running BLAST across multiple cores or CPUs).

#### Visualization

Visualization is a proven invaluable tool in the analysis of complex systems, and the utility of many bioinformatics analyses is enhanced by ready access to good visualization tools. The fundamental properties of biological systems delimit the display dimensions: relationships between biological sequences are tree-like, the sequences themselves can be represented in two dimensions, and their encoded products (protein or RNA) can be represented in three dimensions. Integrative visualization of genotypic and phenotypic properties thus requires the ability to display on sequences, alignments, trees, and three-dimensional (3D) structures diverse types of meta-data such as functional domains, disease association indicators, and statistical estimates. Our approach has been to use the well established ReportLab library [15] and third-party applications such as PyMol [29], where possible. The visualization capabilities of PyCogent discussed below are demonstrated in the Case studies section (below).

For two-dimensional displays, annotation tracks can be associated with alignments or sequences using special pre-defined symbols that show key features such as promoters, untranslated regions, and coding sequences. These symbols are implemented using an extensible DisplayPolicy framework, allowing undesired information to be suppressed and additional user-defined symbols to be added. Fixed and variable types of annotations can be applied to sequence data, with a protein domain illustrating the former and a parameter estimate the latter. These fixed feature types can also be presented on dotplots for comparing different sequences. The capabilities of the ReportLab library have been exploited so that sequence or alignment displays can be sliced and spread over multiple pages to provide additional control over the figure resolution.

Displaying quantitative and qualitative information on phylogenetic trees is still often a stumbling block, with many researchers resorting to exporting the tree topology and then changing colors or line widths after the fact in general purpose graphics packages such as Adobe Illustrator™. PyCogent allows arbitrary callbacks to be used to determine the line color, allowing quantitative information, group labels, or quantitative features related to the sequence or statistical modeling to be displayed directly on the tree (see the second case study, below, for an example of the latter). Additionally, any parameter or combination of parameters can be designated for display on the branches directly.

Similarly, extracting information from a sequence alignment and applying it to a 3D structure determined by crystallography or nuclear magnetic resonance (NMR) has often been performed using *ad hoc *tricks such as overwriting the B-factor column in the PDB file. PyCogent allows arbitrary residues to be colored on a crystal structure, but it also allows quantitative data to be displayed in any of a number of gradients. Additionally, PyCogent allows the sequence from the 3D structure to be extracted and inserted into the alignment, greatly reducing the difficulty typically associated with converting between alignment coordinates and coordinates of the residues that exist in the structure. See the von Willebrand case study (below) for a detailed example of structure coloring.

## Case studies

All code used to conduct the case studies is available in Additional data file 3 as Python scripts, text, and HTML formats.

### A codon aligner and evolutionary analysis of *BRCA1*

By jointly representing the influence of DNA biochemistry and its encoded information on rates of evolution, codon models of sequence change [2,30,31] provide a means for explicitly evaluating the neutral theory of molecular evolution [32]. Under the assumption that synonymous changes are selectively neutral, the ratio of nonsynonymous to synonymous substitution rates (frequently represented as Ka/Ks) is used as an index of natural selection. An assortment of analyses has been developed around these models, motivated by a desire to test for departures from the neutral theory. Briefly, neutrality is violated when there is evidence for Ka/Ks varying temporally (through time, evidenced by different values between tree branches) or when Ka/Ks is significantly greater than 1. Numerous studies have employed these models [3,33,34] and all face the challenge of producing correct sequence alignments before implementing such analyses.

Codon models disallow stop codons and gaps within codons, requiring that indels be positioned respectful of codon boundaries and in multiples of three. The pragmatic solution to producing codon multiple alignments has been translation of DNA sequences into amino acids and alignment of the protein sequences, followed by introduction of indels from the protein sequence alignment into the DNA sequences. PyCogent also provides tools to facilitate this approach. The dynamic programming algorithms for global pair-wise sequence alignment, which are central to progressive multiple alignment, guarantee optimal alignment for the substitution model provided [35]. Codon alignments extrapolated from protein sequence alignments will therefore be optimal for the protein sequences. The only way to ensure codon optimal alignment is to use a codon alignment algorithm.

In PyCogent, different probabilistic analyses are generalized such that they accept any PyCogent substitution model the user creates. For the alignment problem, the user creates a true codon multiple sequence aligner by just providing a codon substitution model. The pair-wise and multiple sequence alignment algorithms implemented in PyCogent are based on the progressive pair-HMMs of Löytynoja and Goldman [36]. Generating a multiple sequence alignment requires a sequence collection, a substitution model for the molecular type, and - optionally - a tree. If no tree is provided, then a neighbor joining tree is built.

**Figure 1:**
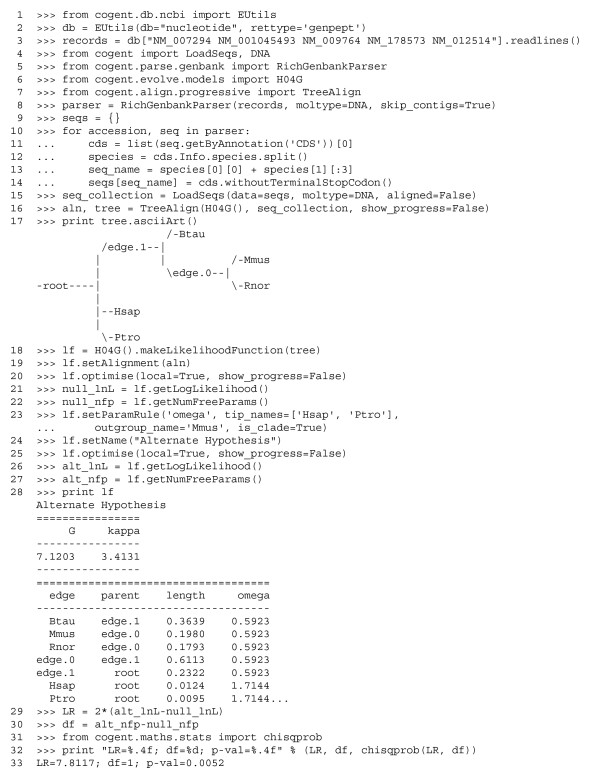
Interactive Python session showing a codon analysis of mammal nucleotide *BRCA1 *sequences. Line numbers are shown at the beginnings of input (but not output) lines and are referenced in the text. The terms '>>>' and '...' represent primary input and continuation prompts, respectively, from a Python interactive session. For noninteractive use, these characters and the following space are removed. The trailing '...' indicates additional output has been truncated.

In this case study, we query the NCBI nucleotide database for specific accessions. We generate sequence names from genus and species, extract the CDS region, eliminate terminal stop codons, and generate a multiple alignment. We then test for evidence of adaptive evolution on the human and chimpanzee lineages. An interactive Python session of the full analysis is shown in Figure 1. In the following, statements regarding line numbers refer to input lines in Figure 1.

We query NCBI for just a set of accessions previously selected for this analysis on the basis of their completeness and inclusion of human and chimpanzee sequences. In order to allow extraction of sequence CDS, we use a parser directly and then remove the terminal stop codon (line numbers 8 to 14), because this state is disallowed by codon substitution models, before aligning. Because long sequence contigs can have multiple features of the same type, it is desirable only to create each segment corresponding to the sought feature type on demand. Having queried NCBI explicitly for *BRCA1 *accession numbers, however, there is only one CDS per sequence and therefore we extract just the first entry in the list (because Python counts from 0, this is the 0th element; line number 11).

As indicated above, the multiple sequence alignment requires a codon substitution model. A number of substitution models are provided for convenience, although we emphasize that a major strength of PyCogent is its flexibility in specifying novel substitution models with minimal effort. For the current case we use the H04G codon substitution model containing terms for transition (labeled in Figure 1 as kappa), nonsynonymous (omega), and CpG (G) substitutions [2]. The aligner returns both the alignment and neighbor joining tree that was used to create it (line 16). We inspect the topology of that tree in an interactive session by printing the text representation generated by the asciiArt method (line 17).

The hypothesis that human and chimpanzee *BRCA1 *genes have evolved in a selectively neutral manner can be evaluated using a hierarchical hypothesis test. In the current example, we explicitly compare the following nested hypotheses: H_o_, the regime of natural selection affecting *BRCA1 *is identical for all lineages (a single Ka/Ks parameter for the entire tree); and H_a_, the selection regime affecting human and chimpanzee *BRCA1 *differs from that affecting the remaining lineages (two Ka/Ks parameters). PyCogent simplifies the formation of such nested hypotheses and significantly eases the task of identifying sections of phylogenetic trees (or tree scopes) to which distinct parameter values are to be applied. Using the tree and same substitution model, we create a likelihood function and provide the alignment (lines 18 and 19). This defines a hypothesis under which all parameters, with the exception of branch lengths, are global across the tree and the probabilities of codons are taken as the average frequency from the alignment, thus completely specifying H_o_. Maximizing the likelihood for this hypothesis given the provided alignment requires numerical optimization. PyCogent provides two different numerical optimizers: simulated annealing [37], which is a global optimizer, and the method proposed by Powell [38], which is a local optimizer whose implementation was derived from the scientific Python library [39]. Although local optimizers typically produce solutions more quickly than do global algorithms, they are vulnerable to being trapped by local optima. The default likelihood function optimizer setting is therefore simulated annealing followed by Powell's method. In most cases, however, the local optimizer suffices and can be explicitly selected using the local argument (line 20). Fine control over all aspects of the optimization is provided by additional arguments for the global and local optimizers. The effect of the optimization is to modify the parameters' values so as to maximize the likelihood for the provided data. The actual log-likelihood and number of free parameters are explicitly extracted (lines 21 and 22).

Definition of the alternate hypothesis requires one to identify the human and chimpanzee tree edges in a robust manner and to associate a distinct value of the parameter omega (which represents Ka/Ks) with that tree scope. The clade is uniquely identified using two tip names, an outgroup name, and setting the argument is_clade = True (line 23). A similar approach can be used to identify just the stem to a clade, or both the clade and its stem. The result of this call is an additional omega value applied to the Hsap and Ptro edges. If we wished to have a different parameter value applied to each edge in the scope, we would set the argument is_independent = True. There is a computational benefit from incrementally building complex hypotheses in this manner; the parameter estimates from optimization of H_o _serve as a starting point for optimization of H_a_, typically resulting in significantly reduced work required for maximization of H_a_. The maximum likelihood (ML) estimates are displayed by printing the lf, indicating omega > 1 for the Hominoid lineages (line 28). We formally test the sufficiency of H_o _by performing a likelihood ratio test using the χ^2 ^distribution with degrees of freedom equal to the difference in the numbers of free parameters between the two hypotheses (lines 29 to 32). In this case, the estimated probability leads us to reject H_o _in favor of H_a_, which is consistent with the previous report of a distinct evolutionary regime affecting *BRCA1 *in humans and chimpanzees [34].

### Visualizing genomic properties in a phylogenetic context for the Proteobacteria

The visual juxtaposition of phenotype and genotype can reveal otherwise obscure patterns in complex data. The distribution of a phenotypic property across a phylogeny is indicative of the rate at which the phenotype evolves and may illuminate its mechanistic origins. Some of the types of questions that can benefit from visualization of phenotypic properties in their phylogenetic context are as follows: the extent to which specific traits are evolvable, the association between ecological niche and evolutionary relatedness, and the distribution of parameter estimates from a statistical analysis.

PyCogent provides multiple tools that allow the user to address such issues easily. These tools include multiple algorithms for phylogenetic tree reconstruction, such as neighbor joining [40] and the tree fit procedures of weighted least squares and ML approaches [41]. A parallelized implementation of the advanced step-wise addition tree space search algorithm [42] is included for use with weighted least squares and ML. PyCogent's generalizations allow any specifiable substitution model to be applied to phylogenetic reconstruction. This ability to use any combination of substitution model with any tree building algorithm allows simple tests for the robustness of a given result to variation in these parameters, addressing a common concern in phylogenetic analyses. Graphical representations of phylogenetic trees can be produced in several standard dendrogram formats. Parameter values for each tree branch can be colored on a spectrum or presented as text. Because the tree labeling or coloring is performed using callbacks that are arbitrary functions of a branch, complex figures that display quantitative data (such as nucleotide composition) or qualitative data (such as group labels, for instance for taxonomic categories or environmental samples) can readily be produced.

We illustrate these capabilities in this case study using a downloaded file of about 5,100 RNA sequences from the rRNA database [43]. We convert these sequences to DNA, sample 30 sequences at random from each of the five taxonomic divisions, exclude near identical sequences, estimate the phylogeny, and draw a radial dendrogram tree with sequence G+C% colored on a spectrum.

**Figure 2:**
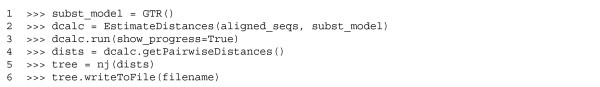
Estimating pair-wise distances. We use a general time reversible nucleotide substitution model (line 1). The pair-wise distances (line 4) are passed to the neighbor joining (nj) function (line 5), which returns a tree that is then written to file (line 6).

The sequence format from this database is for aligned sequences with additional characters representing secondary structure information. For this number of taxa, the reconstruction of an evolutionary tree can prove extremely time consuming. We choose neighbor joining, a distance based phylogenetic method, as the best compromise between accuracy and computational time. The few lines of code required to estimate these distances, generate a neighbor joining tree, and save it to disk are shown in Figure 2. The pair-wise distance estimation code is written to take advantage of multiple CPUs, and the version of the script provided in Additional data file 4 demonstrates the single-line addition (relative to that in Figure 2) necessary to achieve this.

**Figure 3:**
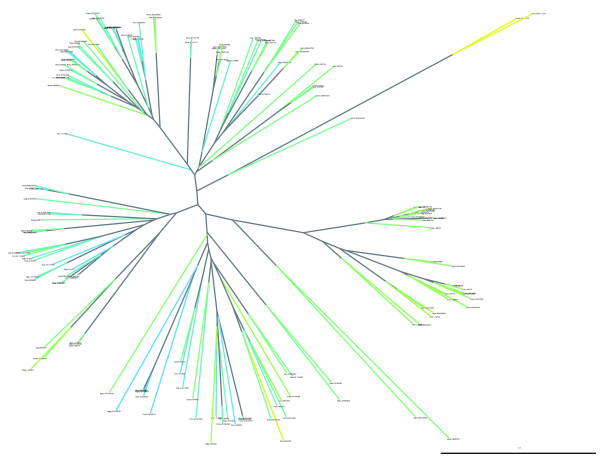
Radial dendrogram displaying Proteobacteria rRNA G+C% on a phylogenetic tree. Low to high G+C% is displayed on a spectrum from yellow to blue. Included are 30 randomly sampled species from each of the five Proteobacteria divisions (α to γ).

To evaluate the phylogenetic distribution of G+C% content, we annotate a tree by setting each sequence's G+C% value in the corresponding tips. Although in the example the G+C% content of ancestral sequences is not determined, internal branches can also be colored. The third-party library ReportLab that PyCogent uses to generate PDF graphics provides fine-grained control over font size, line width, and color. Only two commands are required to produce Figure 3 from the annotated tree. Of interest for this example is the display of G+C% content on a spectrum between the minimum and maximum observed values. We display low G+C% to high G+C% on a spectrum from yellow to blue (note that these colors were chosen so that the spectrum is discernible by red/green colorblind individuals, who comprise an estimated 10% of the male population). We also choose a small font size in order to reduce the amount of visual clutter arising from overlapping tip labels. We note that, in general, closely related taxa exhibit a similar color. However, certain lineages appear to have evolved to low G+C%, quickly raising questions about environmental and/or changes in DNA metabolism that may distinguish these organisms from their sister taxa. Another feature apparent from this tree is a general trend for earlier diverging lineages to be intermediate in G+C%, and for sudden changes toward low G+C% to be more common than sudden changes to high G+C% (there are more yellow branches surrounded by blue or green neighbors than blue branches surrounded by yellow or green neighbors). (Note that the tree drawn is unrooted, but the root based on comparison with other bacterial outgroups is near the center of the displayed tree.) A detailed discussion of these results is beyond the scope of the present work, but the ability of this visualization technique to reveal evolutionary trends should be clear.

### An integrative analysis of von Willebrand factor

In human genetics, distinguishing clinically influential from circumstantial mutations at a candidate locus can be a significant challenge. A number of approaches are used to predict phenotypic impact: linkage studies of the mutation in affected pedigrees; experimental analysis of a mutation on protein function; predicted impact on structure from computer modeling [44]; and an assessment of evolutionary rate [45-47]. The latter approach is based on the hypothesis that a slow evolutionary rate reflects strong negative natural selection caused by functional constraint on a residue. There is increasing interest in drawing on measurement of the historical influence of natural selection to improve predictions regarding the likely phenotypic impact of contemporary variation. Such integration has been demonstrated to improve explanatory power significantly [48].

This case study illustrates an analysis workflow that integrates evolutionary rate classification with molecular structure. We queried the NCBI protein database for von Willebrand Factor (VWF), a 2,813 amino acid glycoprotein that is required for platelet adhesion in blood coagulation. Missense mutations in this molecule have been associated with von Willebrand disease, which is a heterogeneous disorder characterized by prolonged bleeding. We visualize the evolutionary classification of residues in the context of molecular structure and annotated functional domains. We also test whether slowly evolving positions are disproportionately associated with disease.

We query the protein database at NCBI for VWF entries and align the sequences. An inspection of the annotated features column for information regarding disease associated single nucleotide polymorphisms (SNPs) revealed variation in nomenclature, which would increase the complexity of correctly identifying all such variable sites. Records from the Swiss-Prot Protein Knowledgebase, on the other hand, have a consistent notation. We therefore select only full length VWF records from Swiss-Prot using the logical condition 'swissprot' in seq.Info.dbsource. We use annotations on the human record to extract SNP, human disease, and protein domain details using the Info object. Disease status of a SNP is inferred from whether the abbreviation for von Willebrand disease (VWD) occurred in the note field. This query results in only three full length sequences, from human, mouse, and dog. We align the sequences using an empirical protein substitution model [49].

The simpler probabilistic models of sequence evolution assume that all sites have evolved at the same rate throughout time, independently of each other. More realistic evolutionary models have been developed that allow for variation between sites. These models have two major dimensions corresponding to rate (temporal) and spatial heterogeneity. For the temporal component, substitutions are considered to derive from a continuous distribution that has (for computational efficiency) been split into bins. Such models have been applied principally to the model parameter 'rate' and a codon model exchangeability parameter that corresponds to the ratio of nonsynonymous to synonymous substitutions [3]. A Γ distribution is most commonly employed in rate models, although nonregular and mixture distributions have also been specified [50]. Substantial evidence indicates that sequence residues tend to evolve in a manner most similar to their close neighbors, leading to patches of similarly evolving residues. HMMs have been successfully applied to detection of such auto-correlated evolutionary processes [4,51,52]. These phylo-HMMs measure the co-occurrence of residues whose evolution is best described by one of the bins from a rate heterogeneity model. In addition to measuring model fit to a dataset, the classification of sequence residues can be achieved using posterior decoding.

PyCogent allows specification of both classes of model. Rate heterogeneity can be specified as either a Γ or free (nonregular) distribution for rate or any substitution model exchangeability parameter. Although any number of bins can be specified for the rate heterogeneity models, the HMM component at present only allows for two hidden states. If a model with more than two bins is also specified as a HMM, the bins must be mapped to the two hidden states. Although there is strong evidence for spatial heterogeneity of evolutionary rate, a hierarchical hypothesis testing approach should be adopted to validate usage of a phylo-HMM for other parameters.

**Figure 4:**
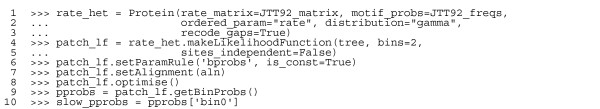
Specifying the phylo-HMM for analysis of VWF. The meaning of the substitution model arguments (lines 1 to 3) are as follows: ordered_param, rate will be split and ordered from small to large across bins; distribution, the statistical distribution by which parameter values are determined; and recode_gaps, whether gap characters are set to 'N'. The substitution model is then turned into a likelihood function (line 5) by providing a phylogenetic tree, specifying that the Γ distribution is split into two bins and the autocorrelated occurrence of rate class members is indicated by the sites_independent argument. We finish the definition of the Γ rate heterogeneity distribution by setting the bin probabilities (bprobs) to be fixed at the default value (line 6), which is equal. The remaining statements provide the alignment data to the likelihood function, optimize it, and extract the posterior probabilities for each site belonging to each rate class (lines 7 to 9). The slow rate class is automatically assigned the name bin0 and those probabilities are extracted by slicing the array (line 10). HMM, hidden Markov model; VWF, von Willebrand Factor.

Returning to our case study, we seek to estimate the probability that each site belongs to the slowly evolving rate class using the multiple sequence alignment. We model sequence evolution using an empirical protein substitution model [49] with rate heterogeneity specified by a Γ distribution with two bins. The auto-correlation of those bins is specified by a phylo-HMM, as described above. The code snippet executing this model and extracting the posterior probabilities is shown in Figure 4. The numerical optimization process used here is simulated annealing [37] followed by the Powell algorithm [38].

**Figure 5:**
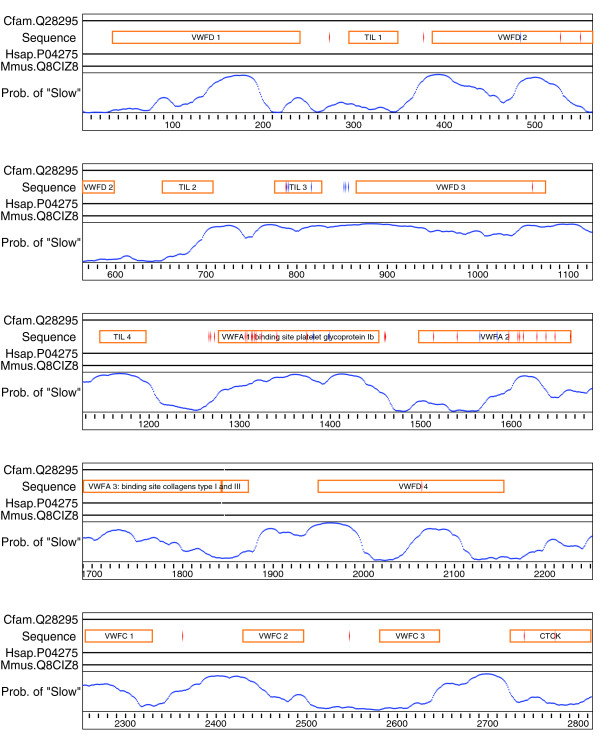
Posterior probabilities of aligned positions being classified as slowly evolving for VWF. Horizontal lines next to each name represent the aligned sequence, with gaps indicated by disruptions to the line (indels disrupt the von Willebrand Factor [VWF] A3 domain). Annotations for a sequence are displayed above its line. Red diamonds are single nucleotide polymorphisms (SNPs) annotated as being associated with von Willebrand disease, blue diamonds are the remaining SNPs. The blue line is the posterior probability a site belongs to the slow (bin0) bin.

Displaying the evolutionary rate classification in direct context with annotated sequence features can be done in two ways. In the first instance, we generate a figure in which the sequence features extracted from the human Swiss-Prot entry are plotted along with the posterior probabilities of the site belonging to the slow (bin0) rate class. These are placed onto either the human sequence, for the annotated features, or the alignment, for the posterior probabilities (Figure 5). Much of the code required to achieve that task is dedicated to splitting the SNPs into disease and nondisease and truncating the labels of the protein domains in order to fit into their corresponding display elements. The plot indicates considerable spatial structure in the occurrence of slow and fast evolving sites, with a clustering of disease associated SNPs in the VWF A1 domain, which is associated with platelet binding.

**Figure 6:**
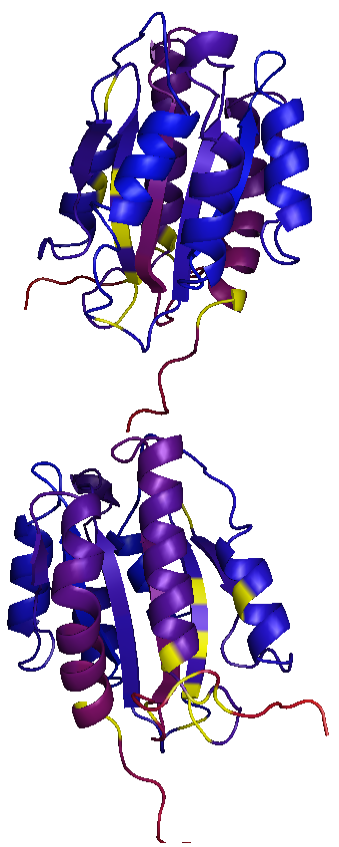
Rates of evolution on the the VWF A1 domain residues. Posterior probabilities of being slowly evolving are shown on a spectrum from red to blue corresponding to low/high probabilities. Residues with a disease causing single nucleotide polymorphism are colored yellow. A movie showing rotation of the structure is provided in Additional data file 3. VWF, von Willebrand Factor.

The next step is to compare the rate categories we estimated from these three sequences with the positions that have been found in other studies to associate with disease in humans. Annotated features such as the posterior probabilities and disease SNP locations can be applied to paint a 3D structure using the third-party PyMol [29] application. We use the structure for the A1 domain [53], as downloaded from PDB (record identifier 1AUQ). Painting the structure requires identification of the portion of sequence covered by the structure, and extraction of the corresponding posterior probabilities and disease SNP locations. The probabilities are displayed as a spectrum scaled from blue (for slowly evolving) to red (fast evolving), with disease SNPs indicated as yellow. We present two snapshots taken using PyMol from opposite sides of the structure (Figure 6) and a movie (Additional data file 2). These suggest a strong relationship between secondary structure features and rates of evolution, and a relationship between 3D proximity of residues and their rate of evolution. PyCogent greatly facilitates the production of this type of figure by extracting the sequence and atom coordinates from the PDB file, relating the PDB sequence to other sequences in a multiple sequence alignment, and performing the coordinate mapping so that properties of the alignment can be applied to residues that exist in the PDB sequence. Because of inherent technical limitations in X-ray crystallography and NMR, PDB records often contain missing residues and multiple chains, and may contain mutations that were introduced to aid analysis (especially truncation of flexible regions and, for RNA, the substitution of short, stable loops). The flexibility in handling missing residues that PyCogent provides is essential for relating evolutionary information across homologs or sequence variants to the available structural data.

We finally evaluate the hypothesis that there is an association between the posterior probability of a position being slowly evolving and there being disease causing variation. PyCogent includes many standard statistical distributions and statistical tests. Summary statistics indicate the mean probability for disease SNPs is 0.58, whereas the mean of the remainder is 0.50. We test whether this difference is significant using PyCogent's implementations of the Kolmogorov-Smirnov test for comparing two distributions. Performing this analysis requires extracting the posterior probabilities for disease SNP locations and for the remainder of the molecule. Running the Kolmogorov-Smirnov test indicates that there are tied values in the data, invalidating the probability estimation. We therefore use the bootstrap version of the KS test, which results in a probability of about 0.2, indicating that the difference in distributions is not significant. Thus, disease-causing SNPs do not occur at sites evolving significantly more slowly than other sites, although these conclusions may change as more full length sequences become available and allow more accurate estimation of the evolutionary rate at each site.

## Conclusion

PyCogent is a unique framework that facilitates the application of comparative genomics data to a broad array of compelling biological problems. The database querying and workflow control capabilities, coupled with a built-in capacity for specifying novel analyses, provide opportunities for comprehensive interrogation of the relationship between genome indexed phenotypic data and evolutionary dynamics. The chosen license, language, and implementation provide a basis for researchers to undertake novel analyses in a manner freed from the release schedule of toolkit developers. PyCogent can be employed in the development of novel methods or standalone applications, to facilitate the integration of new applications and analyses into visual workflow tools such as CIPRES/Kepler [9], and in the development of task centric graphical user interface applications. Several applications have already been developed that draw on PyCogent's capabilities aimed at phylogenetic footprinting [54], analysis of microbial community samples [55,56], combinatorial motif analysis [57], and de-replicating sequence families [58]. The development team welcomes contributions from others who are interested in extending the toolkit to novel problems, integrating it with additional tools and data sources or who seek to exploit it to develop standalone applications.

## Requirements

PyCogent requires the following software: Python 2.5 or greater [59] and Numpy 1.0.3 or greater [14]. Optional dependencies are as follows: ReportLab version 2.0 or greater for visualization [15] (note that this package is required for the case studies presented in this report); Matplotlib version 0.90.1 or greater for visualization of codon usage [16]; PyxMPI version 1.0 or greater for parallel computation [60]; Pyrex for developers who want to modify the *.pyx files [18]; and PyMol version 0.99 or greater for display of 3D structures [29] (note that this package is required for the case studies presented in this report).

## Abbreviations

API, application programming interface; BLAST, basic local alignment search tool; CDS, coding sequence; 3D, three-dimensional; EUtil, Entrez Utility; HMM, hidden Markov model; Ka/Ks, ratio of nonsynonymous to synonymous substitution rates; ML, maximum likelihood; NCBI, National Center for Biotechnology Information; NMR, nuclear magnetic resonance; PDB, Protein Data Bank; Rfam, RNA families database; SNP, single nucleotide polymorphism; VWF, von Willebrand Factor.

## Authors' contributions

A Birmingham, J Carnes, JG Caporaso, M Eaton, M Hamady, H Lindsay, Z Liu, C Lozupone, D McDonald, M Robeson, R Sammut, S Smit, MJ Wakefield, J Widmann, S Wickman, S Wilson and H Ying all contributed source code; B Easton contributed to implementation of matrix algorithms; R Knight, P Maxwell and GA Huttley contributed to overall design and source code; R Knight and GA Huttley are project leaders.

## Additional data files

The following additional data are available with the online version of the paper. Additional data file 1 shows an overview of PyCogent's modules. Additional data file 2 contains a Quicktime movie showing rotation of the painted VWF structure against a black background. Additional data file 3 contains an archive of all scripts used for the case studies presented in this report as Python source, restructured text and HTML formats, and also includes a data file required for the second case study. Additional data file 4 contains an archive of PyCogent version 1.0 source code and documentation.

## Supplementary Material

Additional data file 1 Presented is an overview of PyCogent's modules. Click here for file

Additional data file 2 Presented is a Quicktime movie showing rotation of the painted VWF structure against a black background. Click here for file

Additional data file 3 Presented is an archive of all scripts used for the case studies presented in this report as Python source, restructured text, and HTML formats, as well as a data file required for the second case study. Click here for file

Additional data file 4 Presented is an archive of PyCogent version 1.0 source code and documentation. Click here for file
