# Supplementary material for: PyCogent: a toolkit for making sense from sequence
Source: Genome Biol. 2007 Aug 21;8(8):R171. doi: 10.1186/gb-2007-8-8-r171 (PMC2375001; doi:10.1186/gb-2007-8-8-r171)
Supplement: Additional data file 1 — Presented is an overview of PyCogent's modules. [file gb-2007-8-8-r171-S1.PDF]

Supplementary Material

Primary modules of PyCogent

- Align: code for performing pairwise and multiple sequence alignments, including Pyrex and pure Python implementations of standard algorithms such as Smith-Waterman, Needleman-Wunsch, and pair-HMMs.

- App: controllers for various third-party applications. All applications share a base ApplicationController class, so that adding support for a new application often consists of a few lines of code specifying the parameters and the mechanisms for data input and output. Applications currently wrapped include homology search packages such as BLAST, PSI-BLAST and BLAT, alignment packages such as Clustal and Muscle, motif finders such as MEME, phylogeny packages such as RAxML, and structure analysis packages such as RNAView and the Vienna RNA package.

- Cluster: code for clustering. Currently implements UPGMA and metric scaling.

- Core: the main objects used in many bioinformatics analyeses. The key classes here are Alphabet, Sequence, Alignment, Profile, Tree, Location, MolType, GeneticCode, Info, Bitvector, and Usage.

- Data: bioinformatics data such as energy parameters for RNA folding, and molecular weights of biomolecules.

- Db: interaction with third-party databases, curently NCBI, PDB, and Rfam. A Util module provides generic code for accessing specific databases, including simple lookups in a dict-like interface.

- Draw: Modules for generating publication quality figures. These include dotplots, tree dendrograms, and alignments with annotation tracks.

- Evolve: Probabilistic modelling of sequence evolution. Phylogeny-based maximum-likelihood techniques are implemented such that novel substitution models can be created by the end-user. Continuous time Markov process substitutions models for nucleotide, codon, protein or arbitrary alphabets can be specified that are empirical or parametric. These can be specified as rate-heterogeneous, or phylo-HMM processes. Additional capabilities are sequence simulation and ancestral sequence reconstructions. Temporal dissection of evolutionary processes are also facilitated.

- Format: formatting for writing files to be analyzed in third-party programs. Currently has methods for rewriting alignments and sequences in standard formats, and writing 3D graphics for analysis in the MAGE and PyMol viewers. Also a table formatter for rewriting tables for display or for bulk loading into relational databases such as MySQL or Oracle.

- Maths: Routines for nonlinear optimization, Markov models, geometry, matrix logarithms and exponentials, statistics, etc. Many statistical tests are implemented within PyCogent to provide a consistent API and to reduce dependencies on third-party libraries for simple calculations.

- Motif: Routines for finding and operating on sequence motifs, such as RNA or protein active sites. Will ultimately include code for motif finding; currently, only a simple k-word dictionary-based algorithm is implemented.

- Parse: Parsers for standard sequence, alignment and tree file formats. Formats currently supported include AAIndex, Agilent micorarrays, BLAST, bpseq, Clustal, CUTG, EBI, Fasta, GenBank, GCG, GFF, LocusLink, MacSim, MAGE, MEME, NCBI's taxonomy files, Newick, PAML, PHYLIP, RDB, Rfam, RNAfold, RNAview, the Sprinzl tRNA database, generic 2D tables, TinySeq, generic XML, and UniGene. In addition, an extensive framework for building new parsers is supplied. This framework includes simple options for finding records that are delimited by start lines (such as FASTA) or end lines (such as GenBank), records that consist of specified numbers of lines (such as the output of the

Vienna package programs), records that have subrecords nested by whitespace (such as GenBank), records that have subrecords delimited by line prefixes (such as EBI), etc. This framework greatly facilitates the generation of new parsers, without the maintenance problems arising from using vast, complex regular expressions.

- Phylo: Modules for building and evaluating phylogenetic trees, including construction of distance matrices, neighbor-joining, least-squares and maximum likelihood trees, consensus trees, and metrics for comparing trees to one another.
- Recalculation: Modules for efficient calculation of likelihoods as used by the evolve and align modules. This includes definitions for different types of parameters that may be optimised (ratio's, probabilities, partitions) and valid dimensions across which they can be applied (tree branches, loci, rate bins). Unnecessary recalculations during optimisation of values are avoided through caching of intermediate results where practicable.
- Seqsim: Code for building customized simulations of trees and sequences under different evolutionary models. These include generating random RNA sequences with defined composition and secondary structure, and generating different kinds of random and systematically constructed trees (including per-node changes in the substitution rate matrix).
- Struct: Modules for manipulating 2D and 3D structure. Currently focuses on RNA 2D structures, including several different representations (dot-bracket, tree, pair list, partner array) and manipulations of these structures.
- Util: General utility code, including extensions of the unit testing framework to handle permutations and statistical uncertainty, parallelization code, code for transforming objects and functions into other objects and functions, code for grouping data in different ways for summary reports, array operations, 2-dimensional sparse arrays implemented as dictionaries of dictionaries, code for writing checkpoints to a file, etc.
