# Supplementary material for: PyCogent: a toolkit for making sense from sequence
Source: Genome Biol. 2007 Aug 21;8(8):R171. doi: 10.1186/gb-2007-8-8-r171 (PMC2375001; doi:10.1186/gb-2007-8-8-r171)
Supplement: Additional data file 3 — Presented is an archive of all scripts used for the case studies presented in this report as Python source, restructured text, and HTML formats, as well as a data file required for the second case study. [file gb-2007-8-8-r171-S3.gz › PyCogentPaperComputable/doc/case_studies_index.html]

xml version="1.0" encoding="utf-8" ?


PyCogent Case Studies


# PyCogent Case Studies

Contents

- Simplified Execution of the Case Studies
- Case study 1: BRCA1 evolution
  - Querying NCBI
  - Parsing the GenBank Sequence Records
  - Building a True Codon Aligner
  - Testing for Temporal Heterogeneity in Natural Selection
- Case study 2: Visualizing genomic properties in a phylogenetic context
  - Sampling the sequences
  - Estimating the Pairwise Distances and Building the Neighbor-Joining Tree
  - Visualise Sequence Composition Statistic on the Tree
- Case study 3: Integrative Analysis of von Willebrand Factor
  - Querying NCBI
  - Aligning VWF
  - A phylo-HMM for the analysis of VWF
  - Displaying evolutionary estimates with structural and variation annotations
  - Displaying evolutionary estimates on a crystal structural
  - Testing the relationship between evolutionary rate class and disease

In the PyCogent paper we present 3 case studies. This file shows the actual scripts in all their gory detail, and provides links to the raw restructured text files to generate these html files and the python scripts. The raw restructured text files are computable documents, the lines delimited by >>> and ... are converted and executed by the Python module doctest which is distributed with Python. A script for executing these tests is provided. We note here that this case studies directory contains some empty directories for (e.g. data, figs). Note that absolute dependencies for the complete replication of the analyses are ReportLab and PyMol (see the manuscript for url's). These are used by the case study scripts.

|  |  |
| --- | --- |
| Note: | With the exceptions of a data file for case study 2, all others specified in the script are the result of executing the scripts in the order indicated in the scripts. |

# Simplified Execution of the Case Studies

Separate python files joining the pertinent scripts in the order required for complete execution of each case study are provided in the src/ directory. Specifically, case\_study\_1.py, case\_study\_2.py, case\_study\_3.py.

# Case study 1: BRCA1 evolution

In this case study, we query the NCBI nucleotide database for specific accessions. We generate sequence names from genus and species, extract the CDS region, eliminate terminal stop codons and generate a multiple alignment. We then test for evidence of adaptive evolution on the human and chimpanzee lineages. The script structure presented below is not identical to that from the paper figure, mainly due to placing imports immediately above the statements that require them.

## Querying NCBI

We load the components necessary for querying NCBI and create an EUtils instance pointed at the nucleotide data base and set to return GenBank formatted sequence records.

```
>>> from cogent.db.ncbi import EUtils
>>> db = EUtils(db="nucleotide", rettype="genpept")
>>> records = db["NM_007294 NM_001045493 NM_009764 NM_178573 NM_012514"].readlines()
```

## Parsing the GenBank Sequence Records

PyCogent provides multiple mechanism for loading sequence data. In the current case, we are not loading from an existing file, but the records instance created above behaves like a file that is being read one line at a time. For this example, we are aiming at producing data that can be analysed by a codon substitution model. These models require a DNA sequence that is in frame without any stop codons. For this reason, we selected the GenBank format (above) with feature table, as that table contains location information for the CDS. The RichGenbankParser automatically creates annotation instances (which we refer to as annotation tracks) on each corresponding sequence. These can be extracted in a number of ways, here we use the getByAnnotation method. This returns a generator which allows iteration across all CDS segments in a sequence. In the current case, as we explicitly queried for known accessions there is only 1 CDS per sequence and we therefore use the list() call to create it and then extract the CDS as the 0th list entry.

The other form of sequence annotation is also illustrated below where it is used to create more useful sequence names than just the sequence accessions. The Info object captures all sorts of useful data from the feature table that can be directly accessed as attributes on the sequence. In the current case, we split the species name into words, and form the seq\_name from the first letter of the first word (indexed as [0][0] since Python counts from 0) and the first 3 letters of the second word. This corresponds to the first letter of the genus and first three of the species names. Finally, the 3' terminal stop codons from each sequence are eliminated simply using the withoutTerminalStopCodon method.

```
>>> from cogent import LoadSeqs, DNA
>>> from cogent.parse.genbank import RichGenbankParser
>>> parser = RichGenbankParser(records, moltype=DNA, skip_contigs=True)
>>> seqs = {}
>>> for accession, seq in parser:
...     cds = list(seq.getByAnnotation("CDS"))[0]
...     species = cds.Info.species.split()
...     seq_name = species[0][0] + species[1][:3]
...     seqs[seq_name] = cds.withoutTerminalStopCodon()
>>> seq_collection = LoadSeqs(data=seqs, moltype=DNA, aligned=False)
```

## Building a True Codon Aligner

Typically codon alignments have been produced by translating into amino-acids, aligning the resulting protein sequences and then mapping the indels from that protein sequence alignment back onto the corresponding DNA sequence. *That is not what we do here.* Below we use a progressive pair-HMM aligner that uses a codon substitution model for its score function. We use a canned substitution model and import the progressive alignment function (TreeAlign). This function minimally requires a substitution model and a sequence collection.

If a tree is provided, then the branch length estimates from that tree and default substitution model parameter values will be used. Since those default values are typically 1.0, it is desirable to specify values. This can be done using the TreeAlign functions param\_vals argument.

If no tree is provided, a neighbour-joining tree is computed. The neighbour-joining requires pairwise distances and these are computed using the provided substitution model to: (i) pairwise align the sequences using a pair-HMM; (ii) then estimate the evolutionary distance from the aligned pair. This is the slowest part of the computations but it can be run in parallel across multiple CPU's. The tree estimation process is also used to obtain estimates of the substitution model parameters, the median value from all pairs is provided to the model prior to conducting the progressive alignment. You can see indications of progress by setting show\_progress=True. The aligner also returns the tree topology used. This can be displayed in an interactive Python session by printing the asciiArt result.

```
>>> from cogent.evolve.models import H04G
>>> from cogent.align.progressive import TreeAlign
>>> aln, tree = TreeAlign(H04G(), seq_collection, show_progress=True)

Estimating pairwise distances to build NJ tree
Doing [1/10]: Btau <-> Ptro
    Pairwise aligning: Ptro <-> Btau
Doing [2/10]: Btau <-> Rnor
    Pairwise aligning: Rnor <-> Btau
Doing [3/10]: Btau <-> Hsap
    Pairwise aligning: Hsap <-> Btau...
>>> print tree.asciiArt()
                    /-Btau
          /edge.1--|
         |         |          /-Mmus
         |          \edge.0--|
-root----|                    \-Rnor
         |
         |--Hsap
         |
          \-Ptro
```

## Testing for Temporal Heterogeneity in Natural Selection

We address the hypothesis that the selective regime affecting *BRCA1* is identical for all sampled branches by first specifying a null model in which it is the same. We use the same codon model as was used for the alignment and using the tree result from above we create a likelihood function. We provide the alignment to this function and maximise the likelihood of this hypothesis by optimising. Here we'll print this function (but note the trailing ... indicating the output has been truncated) so we can see what the estimate for the parameters are under this null hypothesis.

```
>>> lf = H04G().makeLikelihoodFunction(tree)
>>> lf.setAlignment(aln)
>>> lf.optimise(local=True, show_progress=False)
>>> lf.setName("Null Hypothesis")
>>> print lf
Null Hypothesis
==========================
     G     kappa     omega
--------------------------
7.1189    3.4122    0.6037
--------------------------
==========================
  edge    parent    length
--------------------------
  Btau    edge.1    0.3635
  Mmus    edge.0    0.1979
  Rnor    edge.0    0.1792
edge.0    edge.1    0.6102
edge.1      root    0.2320
  Hsap      root    0.0124
  Ptro      root    0.0095...
```

The value of omega (~0.6), which represents the ratio of nonsynonymous to synonymous substitutions is < 1.0, indicative of a sequence under the influence of purifying selection. (Note the trailing ... indicates the output has been abbreviated for the purpose of reducing the size of this document.) In order to contrast the support for null and alternate (to be defined below) hypotheses, we need to obtain the log-likelihood and number of free parameters as these are essential for computing the likelihood ratio test (LRT) statistic.

```
>>> null_lnL = lf.getLogLikelihood()
>>> null_nfp = lf.getNumFreeParams()
```

The alternate hypothesis is that the human (Hsap) and chimpanzee (Ptro) lineages evolve in a manner concordant with each other but distinct from the other sampled lineages. We specify this hypothesis by modifying the null using the setParamRule method. The appropriate tree branches are identified using the tip\_names and outgroup\_name arguments, and grouped as a single tree scope by the is\_clade argument. We modify the name and optimise using the local (Powell) numerical optimiser. The statistics required for the LRT are obtained as above.

```
>>> lf.setParamRule("omega", tip_names=["Hsap", "Ptro"], outgroup_name="Mmus", is_clade=True)
>>> lf.setName("Alternate Hypothesis")
>>> lf.optimise(local=True, show_progress=False)
>>> alt_lnL = lf.getLogLikelihood()
>>> alt_nfp = lf.getNumFreeParams()
>>> print lf
Alternate Hypothesis
================
     G     kappa
----------------
7.1203    3.4131
----------------
====================================
  edge    parent    length     omega
------------------------------------
  Btau    edge.1    0.3639    0.5923
  Mmus    edge.0    0.1980    0.5923
  Rnor    edge.0    0.1793    0.5923
edge.0    edge.1    0.6113    0.5923
edge.1      root    0.2322    0.5923
  Hsap      root    0.0124    1.7144
  Ptro      root    0.0095    1.7144...
```

The omega estimates for the Hsap and Ptro lineages are now included in the main display table and are greater than 1. We test whether this hypothesis represents a significant improvement over the null, computing the LR and using PyCogent to compute the probability of observing a larger or equal LR by chance from the chisq distribution.

```
>>> LR = 2*(alt_lnL-null_lnL)
>>> df = alt_nfp-null_nfp
>>> from cogent.maths.stats import chisqprob
>>> print "LR=%.4f; df=%d; p-val=%.4f" % (LR, df, chisqprob(LR, df))
LR=7.8117; df=1; p-val=0.0052
```

The result provides strong support for distinct evolutionary forces affecting human and chimpanzee *BRCA1*.

This analysis can be directly computed using the codon analysis python script.

# Case study 2: Visualizing genomic properties in a phylogenetic context

In this case study we use a downloaded data file of ~5100 RNA sequences from the rRNA database. We convert these sequences to DNA, sample 50 sequences at random from each of the five taxonomic divisions, estimate the phylogeny and draw a radial dendrogram tree with sequence G+C% colored on a spectrum. Most of the complexity below derives from handling the random sampling. The actual reading of the data and conversion of RNA to DNA could be achieved in less than 10 lines.

## Sampling the sequences

This example involves downloading a file for rRNA sequences from the from the rRNA database in distribution format. Selecting the Proteobacteria checkbox, clicking the 'Get sequences' button and saving the resulting large (~40MB) file to disk. This not a simple sequence format, as illustrated by this snippet:

```
acc:AF254109
src:
str:TB66
ta1:Bacteria Proteobacteria {alpha subdivision}
chg:
rem:
aut:Teske A., Brinkhoff T., Muyzer G., Moser D.P., Rethmeier J., Jannasch H.W.
ttl:Diversity of Thiosulfate-Oxidizing Bacteria from Marine Sediments and Hydrothermal Vents
jou:Appl. Environ. Microbiol.
dat:2000
vol:66(8)
pgs:3125-3133
mty:
seq:hydrothermal vent strain TB66 AF254109
--------------------------------------------------------------------------------
--------------------------ooo[oo-ooo]o-----oo[o-ooo]-[o-ooUC]-A[GAA-C{G}AA--CGCU
]---G[GCG-GCA-G-G]C----C-UAA--[CA-C{A--}UGC]-A--A[GUC{-GA--}GC(G)C-(C)CUC]------
```

as the format contains secondary structure information.

Prior to reading in the sequence data we define a utility function that will be used to generate a sequence name. We attempt to use the Genus and species and sequence acession number as its name, but in some cases only a genus host species or description was available. The identification of the genus name is done primitively through use of a regular expression to find capital letters. The function generates a name with form [G]enus [spe]cies and accession to return equivalent of: Gspe.accession; or, if only [Genu]s or only [d]escriptive [phr]ase.

We import Pythons regular expression (re) library.

```
>>> from __future__ import division
>>> import re
>>> def generate_seqname(genus_species, accession=""):
...     genus = re.search("[A-Z]", genus_species)
...     if genus:
...         name = genus_species[genus.start():]
...     else:
...         name = genus_species
...     name = name.split()
...     # some times there is a genus name but no species name
...     if len(name) == 1:
...         name = "%s" % (name[0][:4])
...     # if species name is too short, we'll fill in from genus name
...     elif len(name[1]) < 3:
...         name = "%s" % (name[0][:4])
...     else:
...         name = "%s%s" % (name[0][0], name[1][:3])
...     if accession:
...         accession = ".%s" % accession.split()[-1]
...     return "%s%s" % (name, accession)
```

With this function in place we then import the RdbParser. As we will only analyze a randomly selected subset of the sequences, we create a separate variable (divisions) to store sequences. Each acceptable sequence is appended to the appropriate list.

```
>>> from cogent.parse.rdb import RdbParser
>>> divs = {"alpha":[],"beta":[],"gamma":[],"delta":[],"epsilon":[]}
>>> for data in RdbParser(open("../data/Proteobacteria.txt")):
...     species_accession = data.Info.Species
...     accession = species_accession.split()[-1]
...     species = species_accession.replace(accession, "").strip()
...     data.Name = generate_seqname(species, accession)
...     div = re.findall("(alpha|beta|delta|gamma|epsilon)",
...                            data.Info.Taxonomy1)
...     if div:
...         divs[div[0]].append(data)
```

For the purpose of illustration, we select 30 random sequences from each division. PyCogent provides a generate sequence loading mechanism (LoadSeqs) that requires (name, seq) pairs. Below we use Python's built-in random module to choose the sequences for each division, and load the resulting seqs object as an alignment specifying the MolType as RNA since we will convert to DNA for the tree reconstruction.

|  |  |
| --- | --- |
| Note: | Since the subsequent script can take several hours to run, the execution of a simplified version of the examples can be done by changing 30 below to say 10. |

```
>>> import random
>>> seqs = []
>>> for div in divs:
...     seqs += [(s.Name, s) for s in random.sample(divs[div], 30)]
>>> from cogent import LoadSeqs, RNA
>>> aln = LoadSeqs(data=seqs, moltype=RNA)
```

Most substitution models treat indel characters as N's, so those states, or the full ambiguity characters ('?'), add no information to an analysis. Given the current sequences were derived from a much larger alignment, and there are numerous '?' characters in the alignment, we want to eliminate columns that consist of only the '?' or '-' characters. We use Python's built-in set object to simplify this task. These objects enable set operations (intersection, union, difference) with a simple syntax. We define the lambda function (a special 1 line function) not\_gaps\_or\_missing to return True when the intersection of the set of characters in an alignment column with the set('?-') is not empty.

```
>>> not_gaps_or_missing = lambda col: not set(col) & set("-?")
>>> aln = aln.filtered(not_gaps_or_missing)
```

In sampling the sequences, additional considerations are that we want to eliminate sequences that are too similar to each other. To do this, we define a function that, when passed two sequence objects returns their percent difference for seq1 and seq2, ignoring any aligned column with a gap or missing data.

```
>>> def calc_pc_diff(seq1, seq2):
...     gaps_or_missing = lambda col: set("?-") & set(col)
...     length = len(seq1)
...     seqs = zip(seq1, seq2)
...     num_diffs = 0
...     for col in seqs:
...         if not gaps_or_missing(col):
...             if col[0] != col[1]:
...                 num_diffs += 1
...     return num_diffs/float(length)
```

This function is then used by the Alignment.distanceMatrix method to compute the pairwise distances.

```
>>> dists = aln.distanceMatrix(calc_pc_diff)
```

Before we derive our final sample, we need two more functions. The first is required by the second and, when provided with two sequences returns the name of the shortest sequence after removing ambiguity or gap characters.

```
>>> def name_shortest(seq1, seq2):
...     len_seq1 = len(re.sub("[?-]","",str(seq1)))
...     len_seq2 = len(re.sub("[?-]","",str(seq2)))
...     if len_seq1 < len_seq2:
...         shortest = seq1.Name
...     else:
...         shortest = seq2.Name
...     return shortest
```

This function is used by the select sequence function, which takes the alignment and the pairwise distance matrix and returns an alignment for which all sequences differ by at least 5 nucleotides.

```
>>> def select_seqs(aln, dists):
...     min_pid = 5./len(aln)
...     include = set(aln.Names)
...     for name, name_dists in dists.items():
...         if name not in include: # this seq has been excluded
...             continue
...         for other, dist in name_dists.items():
...             if dist < min_pid and other != name: # no self-self dists
...                 shortest = name_shortest(aln.NamedSeqs[name], aln.NamedSeqs[other])
...                 include.discard(shortest)
...     return aln.takeSeqs(include)
...
>>> aln = select_seqs(aln, dists)
```

The final step is simply the conversion to DNA, which is achieved trivially.

```
>>> aln = aln.toDna()
```

The result of these operations is then written to a fasta formatted file in the data directory.

```
>>> aln.writeToFile("../data/Proteobacteria.fasta")
```

Note that, because we are randomly sampling sequences with this script we will generate a different sample each time and so the results will be different to those shown in the paper.

## Estimating the Pairwise Distances and Building the Neighbor-Joining Tree

This step is the slowest of all the case study procedures (it can take several hours) because of the large size of the data set. It is possible to run the pairwise distance estimation in parallel -- distributing computations across multiple CPU's. To run this script in parallel requires you have both openmpi and PyXMPI installed. Then simply enter $ mpirun -np 2 python build\_rRNA\_tree.py, if you have 2 CPU's for instance.

The actual scripting required is trivial. We load the fasta alignment created in the previous section. To save a bit of typing we define the common name 'Proteobacteria'

```
>>> from cogent import LoadSeqs
>>> name = "Proteobacteria"
>>> aln = LoadSeqs("../data/%s.fasta" % name)
```

To perform the pairwise distance estimation requires a substitution model, and in this case we will use the built-in general time reversible (GTR) model. It also requires an EstimateDistances object, which takes the substitution model and the sequence alignment and is used to perform the distance estimation.

```
>>> from cogent.evolve.models import GTR
>>> from cogent.phylo.distance import EstimateDistances
>>> dcalc = EstimateDistances(aln, GTR())
>>> dcalc.run(show_progress=True, progress_interval=250)
>>> dists = dcalc.getPairwiseDistances()
```

At this point, if we are running a parallel job we bear in mind that we only want a single CPU to write out results. We use the convenience attribute of cogent.util.parallel.output\_cpu for this. This attribute is True on the CPU designated a rank of 0 by mpi. When it is true we import the neighbor-joining function nj and use that to infer the phylogenetic tree which we write to file.

```
>>> from cogent.util.parallel import output_cpu
>>> if output_cpu:
...     from cogent.phylo.nj import nj
...     tree = nj(dists)
...     tree.writeToFile("../results/%s.tree" % name)
```

This analysis can be directly computed using the distance analysis python script.

## Visualise Sequence Composition Statistic on the Tree

To visualise the sequence G+C% on the tree, we first need to compute them. We load the fasta formatted alignment using the LoadSeqs mechanism. We similarly load the tree computed in the previous section using the LoadTree function.

```
>>> from cogent import LoadSeqs, LoadTree
>>> name = "Proteobacteria"
>>> aln = LoadSeqs("../data/%s.fasta" % name)
>>> tree = LoadTree("../results/%s.tree" % name)
```

We determine the G+C% composition of each sequence in the alignment and set it on the tree directly. The values are set on the tree directly using the tree edge name by the setParamValue method. As we want to scale the colour spectrum between the observed extremes of composition we also compute and return those statistics.

```
>>> nums = []
>>> for Name in aln.Names:
...     Seq = aln.getSeq(Name)
...     gc = Seq.count("G") + Seq.count("C")
...     gc /= float(len(Seq))
...     tree.setParamValue("GC", Name, gc)
...     nums.append(gc)
>>> min_GC = min(nums)
>>> max_GC = max(nums)
```

The production of a pdf is simple, but requiring colors and a dendrogram class. For the current case we use the UnrootedDendrogram which produces a radial tree display and create an instance by just passing the modified tree.

```
>>> from cogent.draw.dendrogram import colors, UnrootedDendrogram
>>> np = UnrootedDendrogram(tree)
```

We define a color spectrum of edge and highlight colors that should be visible to both color-blind and normal visioned individuals. These conditions are provided as arguments to the drawToPDF method.

```
>>> height, width = 500, 700
>>> Color = colors.Color
>>> y = Color(1,1,0)
>>> o = Color(.35,.7,.9)
>>> np.drawToPDF("../figs/rRNA-tree.pdf", width, height,
...             stroke_width=1, shade_param = "GC", highlight_color=o,
...             edge_color=y, max_value=max_GC, min_value = min_GC,
...             show_internal_labels=False, font_size = 2,
...             scale_bar = "right")
```

This analysis can be directly computed using the rRNA display analysis python script.

# Case study 3: Integrative Analysis of von Willebrand Factor

In this case study we illustrate an analysis workflow that integrates evolutionary rate classification with molecular structure. We query the NCBI protein data base for von Willebrand Factor (VWF), a 2813 amino acid glycoprotein required for platelet adhesion in blood coagulation. Missense mutations in this molecule have been associated with von Willebrand disease, a heterogeneous disorder characterized by prolonged bleeding. We visualize the evolutionary classification of residues in the context of molecular structure and annotated functional domains. We also test whether slowly evolving positions are disproportionately associated with disease.

## Querying NCBI

As previously, we import EUtils for querying NCBI. In this case, we search the protein data-base and restrict our search to mammal sequences.

```
>>> from cogent.db.ncbi import EUtils
>>> db = EUtils(db="protein", rettype="genpept")
>>> query = '"VWf"[gene] AND Mammalia[orgn]'
>>> records = db[query].readlines()
```

As in the codon sequence analysis case study, we have requested the GenBank record format. Again we use the RichGenbankParser. As we have used a more general query in this case there is greater content diversity in the returned records that we must deal with. For the current case study, we also seek to extract additional data from the feature tables that pertain to the locations of SNPs and whether those SNPs have a disease association. An inspection of the feature tables for human entries reveals that the Swissprot entry had the most complete and accessible content regarding the SNP data: region features with region\_name="Variant" and a note field that contains the indicated amino acid difference along with an indication of whether the SNP was associated with von Willebrand disease (denoted by the symbol VWD). Finally, we seek to extract the protein domain locations for presentation purposes.

We access attributes of the Info object to restrict sequences to Swissprot entries, and extract annotation data from only the human entry. We use regular expressions to assist with extracting data regarding the amino acid change and the domain names. We also name sequences based on their [G]enus [spe]cies and accession.

The selected species are accumulated in a seqs dictionary, keyed by their name. The feature data are accumulated in a list.

```
>>> import re
>>> from cogent.parse.genbank import RichGenbankParser
>>> parser = RichGenbankParser(records)
>>> seqs = {}
>>> feature_table = []
>>> for accession, seq in parser:
...     if "swissprot" not in seq.Info.dbsource or len(seq) < 2800:
...         continue
...     # we extract annotation data only from the human record
...     if "Homo" in seq.Info.species:
...         for feature in seq.Info.features:
...             if "region_name" not in feature:
...                 continue # ignore this one, go to the next feature
...             if "Variant" in feature["region_name"]:
...                 note = feature["note"][0]
...                 variant = " ".join(re.findall("[A-Z] *-> *[A-Z]", note))
...                 disease = "VWD" in note
...                 lo = feature["location"].first() - 1
...                 hi = feature["location"].last()
...                 feature_table.append(["SNP", lo, hi, variant, disease])
...             else:
...                 region_name = feature["region_name"][0]
...                 if region_name == "Domain":
...                     note = [field.strip() for field in re.split("[.;]", feature["note"][0]) if field]
...                     if len(note) == 1:
...                         note += [""]
...                     lo, hi = feature["location"].first() - 1, feature["location"].last()
...                     feature_table.append(["Domain", lo, hi, note, False])
...     species = seq.Info.species.split()
...     seq_name = "%s.%s" % (species[0][0] + species[1][:3], accession)
...     seqs[seq_name] = seq
```

We convert the sequences to a SequenceCollection using LoadSeqs and then save to a fasta formatted file.

```
>>> from cogent import LoadSeqs
>>> seqs = LoadSeqs(data=seqs, aligned=False)
>>> seqs.writeToFile(filename="../data/vwf.prot.fasta")
```

We convert the features into a PyCogent Table object, which requires we specify column headings. We then save to file in Python's native pickle format but note that standard delimited formats (csv or tab) are also possible.

```
>>> from cogent import Table
>>> feature_table = Table(header=["Type", "Start", "Stop", "Note", "Disease"],
...                         rows=feature_table)
>>> feature_table.writeToFile("../data/vwf.prot.features.pickle")
```

## Aligning VWF

We load the previously save unaligned sequences and use an implementation of the Jones Taylor and Thornton empirical substitution model and the built-in progressive-HMM aligner. The TreeAlign function returns both the alignment and the tree that we will save to disk. For the current case we don't suppress the output given the small number of sequences. In the case of the tree, as we only have 3 sequences there is only 1 possible unrooted tree.

```
>>> from cogent import LoadSeqs
>>> from cogent.evolve.models import JTT92
>>> from cogent.align.progressive import TreeAlign
>>> seqs = LoadSeqs("../data/vwf.prot.fasta", aligned=False)
>>> aln, tree = TreeAlign(JTT92(), seqs)

Estimating pairwise distances to build NJ tree
Doing [1/3]: Clup.Q28295 <-> Hsap.P04275
    Pairwise aligning: Clup.Q28295 <-> Hsap.P04275
Doing [2/3]: Clup.Q28295 <-> Mmus.Q8CIZ8
    Pairwise aligning: Clup.Q28295 <-> Mmus.Q8CIZ8
Doing [3/3]: Hsap.P04275 <-> Mmus.Q8CIZ8
    Pairwise aligning: Hsap.P04275 <-> Mmus.Q8CIZ8

Doing progressive alignment
>>> aln.writeToFile("../data/vwf.prot-cogent_aligned.fasta")
>>> tree.writeToFile("../results/vwf.prot.tree")
```

## A phylo-HMM for the analysis of VWF

We load the previously saved alignment and tree.

```
>>> from cogent import LoadSeqs, LoadTree
>>> aln = LoadSeqs("../data/vwf.prot-cogent_aligned.fasta")
>>> tree = LoadTree("../results/vwf.prot.tree")
```

We define a site-heterogeneity substitution model in which rate varies according to the gamma distribution. We then define the corresponding patch (phylo-HMM) likelihood function. For this case we just define a model with 2 bins as the phylo-HMM can only handle 2 hidden states. We build the phylo-HMM model from scratch, basing it on the JTT substitution model. This is done by importing the JTT model matrix and motif probabilities from the canned substitution model module (cogent.evolve.models) and the protein substitution model class. We treat the gap state as missing data (recode\_gaps=True, this is the convention). The ordered\_param argument to the Protein constructor indicates that *rate* is the parameter to vary across bins. The key step in defining this as a phylo-HMM rather than just a site-heterogeneity model is setting the makeLikelihoodFunction argument sites\_independent=False.

```
>>> from cogent.evolve.substitution_model import Protein
>>> from cogent.evolve.models import JTT92_matrix, JTT92_freqs
>>> rate_het = Protein(rate_matrix=JTT92_matrix, motif_probs=JTT92_freqs,
...                     ordered_param="rate", distribution="gamma",
...                     recode_gaps=True)
>>> patch_lf = rate_het.makeLikelihoodFunction(tree, bins=2,
...                     sites_independent=False)
```

We complete the specification of the evolutionary model by setting the bin probabilities (bprobs) equal (the default) and fixed (is\_const=True).

```
>>> patch_lf.setParamRule("bprobs", is_const=True)
```

We provide the alignment data and use the default optimisation profile -- global
(simulated annealing) first followed by local (Powell) optimiser -- to maximise
the likelihood. We display the parameter estimates by just printing the patch\_lf.

```
>>> patch_lf.setAlignment(aln)
>>> patch_lf.optimise(show_progress=False)
>>> print patch_lf
Likelihood Function Table
========================
bin_switch    rate_shape
------------------------
    0.0365        3.6513
------------------------
===============================
       edge    parent    length
-------------------------------
Clup.Q28295      root    0.0837
Mmus.Q8CIZ8      root    0.1227
Hsap.P04275      root    0.0750
-------------------------------
========================
 bin    bprobs      rate
------------------------
bin0    0.5000    0.6477
bin1    0.5000    1.3523
------------------------
===============
motif    mprobs
---------------
    A    0.0767...
```

We extract the posterior probabilities corresponding to the probabilities a sites evolution is most consistent with that of the slow class and save those to file in Python's native pickle format for later annotation of sequences and structures. This is done explicitly in this case by loading the cPickle module, creating the file and dumping the posterior probabilities into it.

```
>>> pprobs = patch_lf.getBinProbs()
>>> slow_pprobs = pprobs["bin0"]
>>> import cPickle
>>> probs_outfile_name = "../results/vwf.prot-slow_rate_patch_spans.pickle"
>>> f = open(probs_outfile_name,"w")
>>> cPickle.dump(slow_pprobs, f)
>>> f.close()
```

## Displaying evolutionary estimates with structural and variation annotations

We first construct a 2D display with the spatial distribution of site classification probabilities presented in context with the alignment and annotated structural domains and SNP locations.

```
>>> from cogent import LoadSeqs, Table
>>> aln = LoadSeqs("../data/vwf.prot-cogent_aligned.fasta")
>>> features = Table(filename="../data/vwf.prot.features.pickle")
```

As these SNP and domain locations were derived from the human sequence, the annotation tracks for them must be re-associated with that sequence. We therefore get that specific sequence instance.

```
>>> human_seq = aln.getSeq("Hsap.P04275")
```

There were two different types of SNPs annotated on the human sequence: those with an indication of disease association (VWD in the feature note field); and those without this indication. As a prelude to distinguishing these SNPs by colour, we filter the features for SNPs and domains. The filter method on Table objects provides a means for selecting table rows conditional on a Python statement being true. The condition, in this case, is assigned as a string containing valid Python syntax to the callback argument.

```
>>> snps = features.filtered(callback="Type == 'SNP'")
>>> domains = features.filtered(callback="Type == 'Domain'")
```

As described in the PyCogent paper, annotation track objects are of two basic types: Feature and Variable. All annotation tracks require a start and end location. The Feature type will be employed here to display the locations of the functional domains and the SNP locations. We deal with SNPs first. We could use the filter method again to identify the disease and non-disease subsets but, to illustrate an alternate approach, use a list comprehension to loop over each snp table row and extract the Start and Stop locations for each SNP.

```
>>> from cogent.core.annotation import Variable, Feature
>>> disease = [(row["Start"],row["Stop"]) for row in snps if row["Disease"]]
>>> non_disease = [(row["Start"],row["Stop"]) for row in snps if not row["Disease"]]
```

We apply the resulting lists of coordinates to the human sequence using the addAnnotation method, specifying a built-in symbol and colour attribute type.

```
>>> human_seq.addAnnotation(Feature, "reddiamond", "Disease SNPs", disease)
reddiamond "Disease SNPs" at [272:273, 376:377, 527:528...
>>> human_seq.addAnnotation(Feature, "bluediamond", "Other SNPs", non_disease)
bluediamond "Other SNPs" at [483:484, 788:789, 790:791...
```

In dealing with the domain locations there is the additional complexity of descriptive material that can be too long to fit into the drawn boxes. We use a simple regular expression to remove the words 'main' and 'for' from the descriptions, and do some additional operations to clean up the text. We again add these to human sequence as a Feature type annotation with symbol, type corresponding to domain.

```
>>> import re
>>> domains = domains.getRawData(["Start", "Stop", "Note"])
>>> for start, stop, note in domains:
...     name, comment = note[0], note[1]
...     if comment:
...         name = ": ".join([name, comment])
...     name = re.sub("main|for","", name)
...     name = " ".join(name.split())
...     human_seq.addAnnotation(Feature, "domain", name, [(start, stop)])
domain "VWFD 1" at [33:240]/2813
domain "TIL 1" at [294:348]/2813
domain "VWFD 2" at [386:598]/2813
domain "TIL 2" at [651:707]/2813
domain "TIL 3" at [775:827]/2813
domain "VWFD 3" at [865:1074]/2813
domain "TIL 4" at [1145:1196]/2813
domain "VWFA 1: binding site platelet glycoprotein Ib" at [1276:1453]/2813
domain "VWFA 2" at [1497:1665]/2813
domain "VWFA 3: binding site collagens type I and III" at [1690:1871]/2813
domain "VWFD 4" at [1948:2153]/2813
domain "VWFC 1" at [2254:2328]/2813
domain "VWFC 2" at [2428:2495]/2813
domain "VWFC 3" at [2579:2645]/2813
domain "CTCK" at [2723:2812]/2813
```

We now load the posterior probabilities generated from application of the phylo-HMM. These will be applied to alignment as a Variable annotation type, requiring we create the start, end locations for each alignment position along with the corresponding y-value (corresponding in this case to the posterior probability a site belongs to the slow rate bin). The probabilities will be plotted as a Variable blue line with label Prob of slow.

```
>>> import cPickle
>>> probs_filename = "../results/vwf.prot-slow_rate_patch_spans.pickle"
>>> slow_pprobs = cPickle.load(open(probs_filename))
>>> spans = [([i,i+1], p) for i, p in enumerate(slow_pprobs)]
>>> aln.addAnnotation(Variable, "blueline", 'Prob. of "Slow"', spans)
blueline "Prob. of "Slow"" at [0:2813]/2813
```

This completes the creation of the annotated sequences and alignment required for display. To actually generate the display requires using ReportLab components since the length of VWF is extensive and we would have poor resolution where we to generate a display with the entire result on a single row. We therefore load the SimpleDocTemplate class and page size (A4) to allow us to directly construct the display on a row by row basis. First we create the document, specifying page size, margins and pdf filename.

```
>>> from reportlab.platypus import SimpleDocTemplate
>>> from reportlab.lib.pagesizes import A4
>>> pdf_name = "../figs/vwf.prot-align_patch_display.pdf"
>>> doc = SimpleDocTemplate(pdf_name, leftMargin=10, rightMargin=10, pagesize=A4)
```

The PyCogent annotation tracks on Alignment and Sequence classes are sliced when their sequences are. The same is true for the Display class, which interprets the sequence annotations and converts them into ReportLab objects. We therefore want to define the number of sequence motifs per row (set below as rowlen) and build the figure one row at a time. These slices are converted into ReportLab drawings and accumulated as a story which is then written by the ReportLab doc instance into the pdf file (being careful to append any remainder after the loop).

```
>>> from cogent.draw import Display
>>> disp = Display(aln, min_feature_height=18)
>>> story = []
>>> WIDTH = A4[0]-50
>>> rowlen = 563
>>> start = 0
>>> for end in range(rowlen, len(aln), rowlen):
...     story.append(disp[start:end].asDrawing(total_width=WIDTH,
...     rowlen=rowlen))
...     start = end
>>> story.append(disp[start:len(aln)].asDrawing(total_width=WIDTH,
...     rowlen=rowlen))
>>> doc.build(story)
```

## Displaying evolutionary estimates on a crystal structural

Here we download the crystal structure for PDB id 1AUQ using PyCogent and save it to a file for usage by other computations and for subsequent visualisation with PyMol.

```
>>> from cogent.db.pdb import Pdb
>>> pdb = Pdb()
>>> structure = pdb["1AUQ"].read()
>>> outfile = open("../figs/1AUQ.pdb", "w")
>>> outfile.writelines(structure)
>>> outfile.close()
```

Displaying evolutionary information regarding the rate of site evolution on the structure will be done using a colour spectrum of fast (minimum posterior probability of slow=0.0, red) to slow (maximum posterior probability of slow=1.0, blue). We achieve this by breaking the spectrum up into 20 different bins and creating a distinct colour for each bin. As we also want to annotate the residues at which there is a recorded disease causing variant, we create a special colour outside the spectrum for that purpose.

```
>>> num_colours = 20
>>> def make_colour_spectrum():
...     g = [0.08] * num_colours # for color-blinds
...     b = [float(i)/num_colours for i in range(num_colours)]
...     r = [float(i)/num_colours for i in range(num_colours, 0, -1)]
...     colours = zip(r,g,b)
...     names = ["color_%d" % (i+1) for i in range(num_colours)]
...     return dict(zip(names, colours))
>>> color_map = make_colour_spectrum()
>>> color_map["color_disease"] = (1.,1.,0.)
>>> bins = [(float(i)/num_colours, float(i+1)/num_colours) for i in range(num_colours)]
```

For convenience we create a variable containing the PDB id.

```
>>> pdb_id = "1AUQ"
```

In order to colour the structure we need to determine the portion of the sequence covered by the structure. We do this by pairwise aligning (using the JTT92 model) the human sequence with the sequence chains extracted from the PDB file. As there can be multiple such chains so we loop over them, yielding the chain name, the PDB indicated start position and the indices that correspond to a non-gap match between the two sequences.

```
>>> from cogent import LoadSeqs, LoadTree, Table
>>> from cogent.evolve.models import JTT92
>>> from cogent.align.progressive import TreeAlign
>>> from cogent.format.pdb_color import get_chains, chains_to_seqs,\
...                                     ungapped_to_pdb_numbers
>>> def aln_chain_intersects(pdb_id):
...     vwf = LoadSeqs(filename="../data/vwf.prot-cogent_aligned.fasta")
...     human_seq = vwf.getSeq("Hsap.P04275")
...     tree = LoadTree(treestring="(Hsap, pdb)")
...     chains = get_chains(open("../figs/%s.pdb" % pdb_id))
...     data, seq_type = chains_to_seqs(chains)
...     for chain in chains:
...         struct_seq = data[chain]
...         # we need the start index of the structure's residues
...         index_map = ungapped_to_pdb_numbers(chains[chain])
...         pdb_start = index_map[0]
...         # load these sequences, and determine where the pdb and human
...         # data align
...         seqs = LoadSeqs(data=dict(Hsap=str(human_seq), pdb=struct_seq),
...                         aligned=False)
...         # pair-HMM alignment using TreeAlign
...         aln, tree = TreeAlign(JTT92(), seqs, show_progress=False)
...         indices = aln.getPositionIndices(lambda col: "-" not in col)
...         yield chain, pdb_start, indices
```

The next step towards painting the structure is to load the posterior probabilities and feature table and extract those that actually intersect with the chains included in the structure. We'll use Python's built-in set object, which allows for set-like operations (union, intersection etc.) to determine the appropriate observations for a specific chain. We use the numpy module's take function to succinctly extract the desired data.

```
>>> import cPickle
>>> import numpy
>>> def get_pprobs_snps_for_structure(pdb_id):
...     # load disease SNP indices
...     features = Table(filename="../data/vwf.prot.features.pickle")
...     snps = features.filtered(callback="Type == 'SNP' and Disease")
...     snps = set(snps.getRawData("Start"))
...     # load and index posterior probs from pdb_start
...     pprobs = cPickle.load(open("../results/vwf.prot-slow_rate_patch_spans.pickle"))
...     indexed_pprobs = []
...     indexed_snps = []
...     for chain, pdb_start, indices in aln_chain_intersects(pdb_id):
...         align_start = indices[0]
...         indices = set(indices)
...         # get intersection of disease SNPs and structure, and turn it
...         # into a list
...         snp_indices = list(indices & snps)
...         # get the indices that are not SNP locations
...         not_snp_indices = list(indices - snps)
...         num = len(not_snp_indices)
...         # get the corresponding pprobs
...         pprob_vals = numpy.take(pprobs, not_snp_indices)
...         pprobs_indices_vals = zip(not_snp_indices, pprob_vals)
...         # make a data structure consisting of positions in PDB struct,
...         # pprob and chain
...         for index, val in pprobs_indices_vals:
...             indexed_pprobs += [[index-align_start+pdb_start,val,chain]]
...         # now make the snp indices
...         for index in snp_indices:
...             indexed_snps += [[index-align_start + pdb_start, chain]]
...     return indexed_pprobs, indexed_snps
>>> pprobs, disease_snps = get_pprobs_snps_for_structure(pdb_id)
```

Now that we have the probability indices we want to assign colours along the spectrum based on their values. We define a function to return the colour bin index for a given probability. This function is used by another that constructs Python data objects that represent the colour command list that include the PDB chain label and by-residue colours.

```
>>> def colour_index(val):
...     for index, (start, end) in enumerate(bins):
...         if start <= val < end:
...             return index
>>> def make_color_command_list(pprobs, disease_snps):
...     # each colour has a series of positions assigned to it
...     # disease SNPs are assigned the colour yellow
...     chains = {}
...     for pos, pprob, chain in pprobs:
...         index = colour_index(pprob)
...         if chain in chains:
...             if index in chains[chain]:
...                 chains[chain][index].append(pos)
...             else:
...                 chains[chain][index] = [pos]
...         else:
...             chains[chain] = {index: [pos]}
...     # we turn the positions into strings
...     for chain, color_command in chains.items():
...         for colour, positions in color_command.items():
...             name = "color_%d" % (colour + 1)
...             color_command[name] = "+".join([str(p) for p in positions])
...             color_command.pop(colour)
...     color_command = []
...     for chain in chains:
...         color_command += [([name], [pos], chain) for name, pos in chains[chain].items()]
...     # now add the disease snp coords as yellow
...     if disease_snps:
...         dis_snps = {}
...         for snp, chain in disease_snps:
...             snp = str(snp)
...             dis_snps[chain] = dis_snps.get(chain, []) + [snp]
...         disease_snps = [(["color_disease"],
...                     ["+".join(dis_snps[chain])], chain) for chain in dis_snps]
...         color_command += disease_snps
...     return color_command
>>> color_command_list = make_color_command_list(pprobs, disease_snps)
```

These are then written into PyMol compatible files in working\_dir (defined below), one of which says 'double\_click\_me'. As PyMol is a python based application, Python syntax is written to that file. To complete this, we first import key text necessary for generating the PyMol files and create the necessary strings.

```
>>> import os
>>> from cogent.format.pdb_color import PYMOL_FUNCTION_STRING, MAIN_FUNCTION_STRING
>>> _run_script_string = \
... '''
... from pymol import cmd
... cmd.load("%s")
... cmd.do("run %s")
... '''
>>> _color_function_string = \
... '''
... color_map = %s
... color_command_list = %s
... sticks_command_list = %s
... set_color_list(list(color_map.items()))
... for color_cmd in color_command_list:
...     colors,indices,chain_id = color_cmd
...     set_seq_colors(colors,indices,chain_id)
... for sticks_cmd in sticks_command_list:
...     indices,chain_id = sticks_cmd
...     set_show_shapes(indices,chain_id)
... cmd.bg_color('white')
... '''
>>>
```

We then specify the directory where these PyMol files will be written and populate these strings with color\_map and color\_command\_list that we created above.

```
>>> working_dir = "../figs"
>>> pdb_file = "%s.pdb" % pdb_id
>>> pymol_script_list = [PYMOL_FUNCTION_STRING,MAIN_FUNCTION_STRING]
>>> pymol_script_list.append(_color_function_string % (color_map,\
...                         color_command_list,[]))
>>> pymol_script_string = "".join(pymol_script_list)
>>> pymol_script_name = "%s_motif_coloring.pml" % (pdb_id)
>>> pymol_script_out = open(os.path.join(working_dir, pymol_script_name),'w')
>>> pymol_script_out.write(pymol_script_string)
>>> pymol_script_out.close()
>>> pymol_execute_name = "%s_double_click_me.pml" % (pdb_id)
>>> pymol_execute_string = _run_script_string % (pdb_file, pymol_script_name)
>>> pymol_execute_out = open(os.path.join(working_dir, pymol_execute_name),'w')
>>> pymol_execute_out.write(pymol_execute_string)
>>> pymol_execute_out.close()
```

Completing this visualisation requires installation of PyMol. You can double click the file. Note that the structure can be made to rotate within PyMol using the commands:

```
mset 1 x400
util.mroll(1,400,1)
mplay
```

The command mstop causes the rotation to stop.

The vwf painting script contains the raw python code to execute the above.

## Testing the relationship between evolutionary rate class and disease

We wish to test whether SNPs that cause disease have a greater chance of occurring at residues that are classified as conserved than otherwise. We do this by comparing the distribution of probabilities a site belongs to the slow class from the disease SNP associated positions with those of the remainder using the Kolmogorov-Smirnov (KS) test.

We load the SNP locations from the saved feature table and extract the indices of the disease associated SNPs.

```
>>> from cogent import Table
>>> features = Table(filename="../data/vwf.prot.features.pickle")
>>> snps = features.filtered(callback="Type == 'SNP'")
>>> disease_snp_indices = [row["Start"] for row in snps if row["Disease"]]
```

We then load the posterior probability estimates.

```
>>> import cPickle, numpy
>>> pprobs = cPickle.load(open("../results/vwf.prot-slow_rate_patch_spans.pickle","r"))
```

As we seek to test whether locations with disease associated SNPs tend to be slowly evolving we need to identify the indices for those sites and the indices from the remainder. We use Python's build-in set object for obtaining the positions and then use the numpy.take function for extracting the posterior probabilities corresponding to those positions.

```
>>> pprob_indices = set(range(len(pprobs)))
>>> remaining_indices = pprob_indices.symmetric_difference(set(disease_snp_indices))
>>> remaining_indices = list(remaining_indices)
>>> remaining_indices.sort()
>>> disease_site_probs = numpy.take(pprobs, disease_snp_indices)
>>> remaining_probs = numpy.take(pprobs, remaining_indices)
```

PyCogent can perform different types of statistical tests and also provides convenience classes for dealing with numbers. We use the Numbers class here to generate summary statistics for the two different site-types.

```
>>> from cogent.maths.stats.util import Numbers
>>> print Numbers(disease_site_probs).summarize()
============================
        Statistic      Value
----------------------------
            Count         38
              Sum    22.0487
           Median     0.5644
             Mean     0.5802
StandardDeviation     0.2737
         Variance     0.0749
----------------------------
>>> print Numbers(remaining_probs).summarize()
==============================
        Statistic        Value
------------------------------
            Count         2778
              Sum    1409.9100
           Median       0.5330
             Mean       0.5075
StandardDeviation       0.3283
         Variance       0.1078
------------------------------
```

The summary statistics show that both the mean and median values for the positions at which SNPs with a von Willebrand disease (VWD) association are greater than the remaining positions. This is consistent with the hypothesis that disease variation tends to occur at evolutionarily conserved positions. To explicitly test the differences between the observations we import functions for performing the KS test. As is evident below, the two distributions have tied observations, invalidating the standard version of the KS test. We therefore use the bootstrap variant of the KS test.

```
>>> from cogent.maths.stats.test import ks_test, ks_boot
>>> print ks_test(disease_site_probs, remaining_probs)
(0.169830624076, 0.229736526337)
>>> print ks_boot(disease_site_probs, remaining_probs)
(0.16...
```

The bootstrap variant doesn't substantially affect the estimated probability hence we fail to reject the null hypothesis that the positions at which mutations causing VWD have a greater tendency to belong to the conserved site-class. (Note we truncate the display of the resulting probability as it is sensitive to replicate size and can cause the doctest to fail.)

The vwf KS test script contains the raw Python code.
