# Supplementary material for: PyCogent: a toolkit for making sense from sequence
Source: Genome Biol. 2007 Aug 21;8(8):R171. doi: 10.1186/gb-2007-8-8-r171 (PMC2375001; doi:10.1186/gb-2007-8-8-r171)
Supplement: Additional data file 4 — Presented is an archive of PyCogent version 1.0 source code and documentation. [file gb-2007-8-8-r171-S4.gz › PyCogent/doc/DisplayPolicy_key.pdf]

**Gene Structure**					
misc_RNA		precursor_RNA		prim_transcript	
3'clip		5'clip		mRNA	
exon		intron		3'UTR	
5'UTR		CDS		mat_peptide	
sig_peptide		transit_peptide		polyA_signal	
polyA_site		gene	gene gene	operon	operon operon
**Regulation**					
attenuator		enhancer		CAAT_signal	
TATA_signal		promoter		GC_signal	
protein_bind		misc_binding		-10_signal	
-35_signal		terminator		misc_signal	
rep_origin		RBS			
**Repeats**					
repeat_region		repeat_unit		LTR	
satellite		stem_loop		misc_structure	
**Rna Genes**					
rRNA	rRNA rRNA	scRNA	scRNA scRNA	snRNA	snRNA snRNA
snoRNA	snoRNA snoRNA	tRNA	tRNA tRNA		
**Sequence**					
source	source source	misc_recomb	misc_recomb	variation	variation variation
misc_feature	misc_feature	old_sequence		unsure	
misc_difference		conflict		modified_base	
primer_bind	primer_bind	STS		gap	
**Graphs**					
